# Structures and reactivity of peroxy radicals and dimeric products revealed by online tandem mass spectrometry

**DOI:** 10.1038/s41467-020-20532-2

**Published:** 2021-01-12

**Authors:** Sophie Tomaz, Dongyu Wang, Nicolás Zabalegui, Dandan Li, Houssni Lamkaddam, Franziska Bachmeier, Alexander Vogel, María Eugenia Monge, Sébastien Perrier, Urs Baltensperger, Christian George, Matti Rissanen, Mikael Ehn, Imad El Haddad, Matthieu Riva

**Affiliations:** 1 Univ Lyon, Université Claude Bernard Lyon 1, CNRS, IRCELYON, 69626 Villeurbanne, France; 2 Laboratory of Atmospheric Chemistry, Paul Scherrer Institute, 5232 Villigen, Switzerland; 3 Centro de Investigaciones en Bionanociencias (CIBION), Consejo Nacional de Investigaciones Científicas y Técnicas (CONICET), Godoy Cruz 2390, C1425FQD Ciudad de Buenos Aires, Argentina; 4 Departamento de Química Inorgánica, Analítica y Química Física, Facultad de Ciencias Exactas y Naturales, Universidad de Buenos Aires, Ciudad Universitaria, C1428EGA Buenos Aires, Argentina; 5 Institute for Atmospheric and Environmental Sciences, Goethe-University Frankfurt, 60438 Frankfurt am Main, Germany; 6 Institute for Atmospheric and Earth System Research, INAR /Physics, Faculty of Science, University of Helsinki, FI–00014 Helsinki, Finland; 7 Aerosol Physics Laboratory, Physics Unit, Faculty of Engineering and Natural Sciences, Tampere University, FI-33101 Tampere, Finland

**Keywords:** Atmospheric chemistry, Atmospheric chemistry, Mass spectrometry

## Abstract

Organic peroxy radicals (RO_2_) play a pivotal role in the degradation of hydrocarbons. The autoxidation of atmospheric RO_2_ radicals produces highly oxygenated organic molecules (HOMs), including low-volatility ROOR dimers formed by bimolecular RO_2_ + RO_2_ reactions. HOMs can initiate and greatly contribute to the formation and growth of atmospheric particles. As a result, HOMs have far-reaching health and climate implications. Nevertheless, the structures and formation mechanism of RO_2_ radicals and HOMs remain elusive. Here, we present the in-situ characterization of RO_2_ and dimer structure in the gas-phase, using online tandem mass spectrometry analyses. In this study, we constrain the structures and formation pathway of several HOM-RO_2_ radicals and dimers produced from monoterpene ozonolysis, a prominent atmospheric oxidation process. In addition to providing insights into atmospheric HOM chemistry, this study debuts online tandem MS analyses as a unique approach for the chemical characterization of reactive compounds, e.g., organic radicals.

## Abstract (web summary)

Organic peroxy radicals play a pivotal role in producing highly oxygenated organic molecules but the formation mechanisms remain elusive. Here, the authors show in-situ characterization of peroxy radicals and dimer structures in the gas-phase, using online tandem mass spectrometry analyses.

## Introduction

Atmospheric aerosols adversely affect human health^1^ and have important climate effects. They impact Earth’s radiative balance by directly interacting with light, or indirectly by acting as cloud condensation nuclei (CCN)^2,3^. Their effects on cloud formation and physical properties remain one of the most important uncertainties in climate models^3,4^. Up to 50% of global CCN formation could be attributed to new particle formation (NPF)^5–7^. Rapidly and widely formed, highly oxygenated organic molecules (HOMs)^8–11^ have been identified to be critical for NPF and growth, and therefore for CCN formation^12–15^.

HOM formation proceeds via autoxidation of the peroxy radical (RO_2_), which undergoes intramolecular hydrogen abstraction (H-shift), yielding an alkyl radical (R) with a hydroperoxyl functional group (–OOH). Addition of O_2_ to R produces a new RO_2_ radical, which may undergo termination reactions or further autoxidation reactions^8,16,17^. Autoxidation can therefore yield a plethora of multifunctional products with low or extremely low saturation vapor pressures within seconds^10,18,19^. Studies have also reported rapid gas-phase formation of dimeric accretion products (i.e., organic peroxides, ROOR) produced from RO_2_ + RO_2_ reactions^8,16,20,21^, which can initiate and contribute to NPF^22^ and particle growth^20,23^. However, little is known about the molecular structures of the gas-phase HOMs, including RO_2_ and ROOR, making their formation pathways uncertain. HOMs’ contribution to NPF is directly dependent on their volatility, which can vary by orders of magnitude for isomeric compounds with different structures and functional groups^24,25^.

While quantum chemical calculations and online high-resolution mass spectrometry (MS) analyses using isotopic labeling and deuterium-hydrogen exchange have provided valuable information on HOMs, direct assessment of their molecular structures remains elusive^26–29^. Existing online tandem MS (MS/MS) studies on low-volatility compounds are restricted to the aerosol phase^30–32^. Offline analysis requires sample collection and extensive sample preparation^33^, which can be prone to artifacts from chemical degradation and contamination^34^. Nevertheless, MS/MS measurements in the particle phase^30,35–37^ have provided precious insights into the structure of stable compounds but also unstable gas-phase precursors, for example the identification of stable ester dimers produced from α-pinene ozonolysis^36,38^. α-Pinene is one of the most abundant biogenic volatile organic compounds in the atmosphere, with global emission reaching up to 66.1 Tg yr^−1^, and one of the key precursors for organic aerosol^39^. Similarly, the ozonolysis of limonene (structural isomer of α-pinene) is also expected to play a major role in NPF, despite the lower emission rate (11.4 Tg yr^−1^), due to its greater reactivity toward ozone and a higher HOM yield than α-pinene^10^. In addition, limonene is found in cleaning and personal care products, and might be a significant source for indoor secondary organic aerosols (SOAs)^40–43^. Limonene has a single cyclic structure as opposed to the bicyclic structure of α-pinene, making it a model compound for the elucidation of monoterpene oxidation mechanisms due to the comparatively simple product structures.

In this study, we report the in-situ structural characterization of gas-phase HOMs, RO_2_ radicals, and dimeric products of α-pinene and limonene ozonolysis based on online MS/MS using an ultrahigh-resolution Orbitrap mass spectrometer equipped with a NO_3_^–^ chemical ionization (CI) source^44,45^. Using online MS/MS data, aided by known structure-specific fragmentation patterns from existing offline MS/MS literature, we are able to infer the most plausible isomers of different compounds. We propose the most plausible oxidation product structures, autoxidation mechanisms, and dimer formation pathways based on the observed MS/MS product ions and neutral losses for the two monoterpene precursors.

## Results

### Online MS/MS

**Fig. 1:**
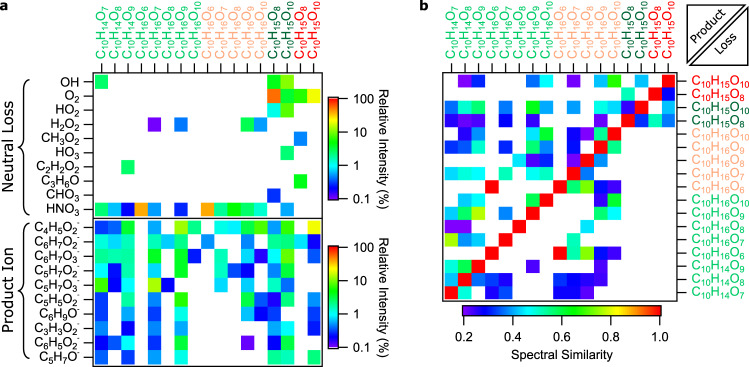
Common MS/MS product ions and neutral losses observed. **a** Selected neutral loss patterns deduced from the formulae of the parent and product ions, and the ten most common product ions observed. **b** Pair-wise spectral similarities in MS/MS spectra. Corresponding MS/MS data were obtained at NCE 2 for NO_3_^–^ adducts of limonene and α-pinene ozonolysis products. Parent molecular formulae are shown on the upper *x*-axis with labels for closed-shell limonene and α-pinene ozonolysis products in light green and light red, respectively. Labels for limonene and α-pinene RO_2_ radical are in dark green and dark red, respectively. Inferred neutral losses and observed product ions with relative intensity ≥0.1% are shown in log color in **a**. The upper triangle and lower triangle areas in **b** represent the cosine similarity between the observed product ions or neutral losses, respectively, among the MS/MS spectra. For clarity, only pairs with cosine similarity ≥0.2 are shown in linear color scale. Parent molecular formulae are also shown on the right *y*-axis in **b**.

Representative mass spectra of limonene and α-pinene ozonolysis products are shown in Supplementary Fig. 1. Online MS/MS was performed on NO_3_^–^ adducts of C_10_H_14–16_O_6–10_ monomers, C_10_H_15_O_6,__8,10_ RO_2_ radicals, and C_18–20_H_28–34_O_6–16_ dimers at different collision energies (normalized collision energy (NCE) from 2 to 10). Overall, as shown for C_10_H_14_O_7_NO_3_^–^ and C_20_H_30_O_14_NO_3_^–^ in Supplementary Figs. 2 and 3, respectively, MS/MS spectrum exhibits a higher signal intensity corresponding to the precursor ion at lower collision energies, while product ions and NO_3_^–^ (declustered CI reagent ion) dominate at higher collision energies. In general, dimers appear to bind more strongly to NO_3_^–^ than do monomers (Supplementary Figs. 2c and 3c)^44^. In Fig. 1, we present an overview of most important neutral losses and product ions observed for HOMs. We infer the neutral loss pattern from the precursor and product ion formula (Fig. 1b). For closed-shell molecules, we observe a common fragmentation pattern involving HNO_3_ loss, which is rare or insignificant for RO_2_ radicals (Fig. 1a). We report the fragmentation patterns of RO_2_ radicals (e.g., C_10_H_15_O_8,10_), all of which undergo O_2_ loss, corresponding to the loss of the peroxy functional group, as shown in Fig. 1a. NO_3_^–^ product ion is observed in all MS/MS spectra. While RO_2_ radicals and closed-shell molecules share some common MS/MS fragments, predominately C_3–6_ ions (Fig. 1a), they exhibit distinct neutral loss patterns and share little spectral similarity (Supplementary Fig. 4b). In general, compounds produced from the same (VOC + O_3_) reactions share spectral similarity with each other (Fig. 1b), which is also reflected in the unsupervised agglomerative hierarchical clustering results (Supplementary Fig. 4a). As discussed below, the neutral loss patterns such as OH, HO_2_, CH_3_O_2_, and C_3_H_6_O observed for RO_2_ radicals (Fig. 1a) are indicative of specific functional groups.

### Autoxidation mechanism

**Fig. 2:**
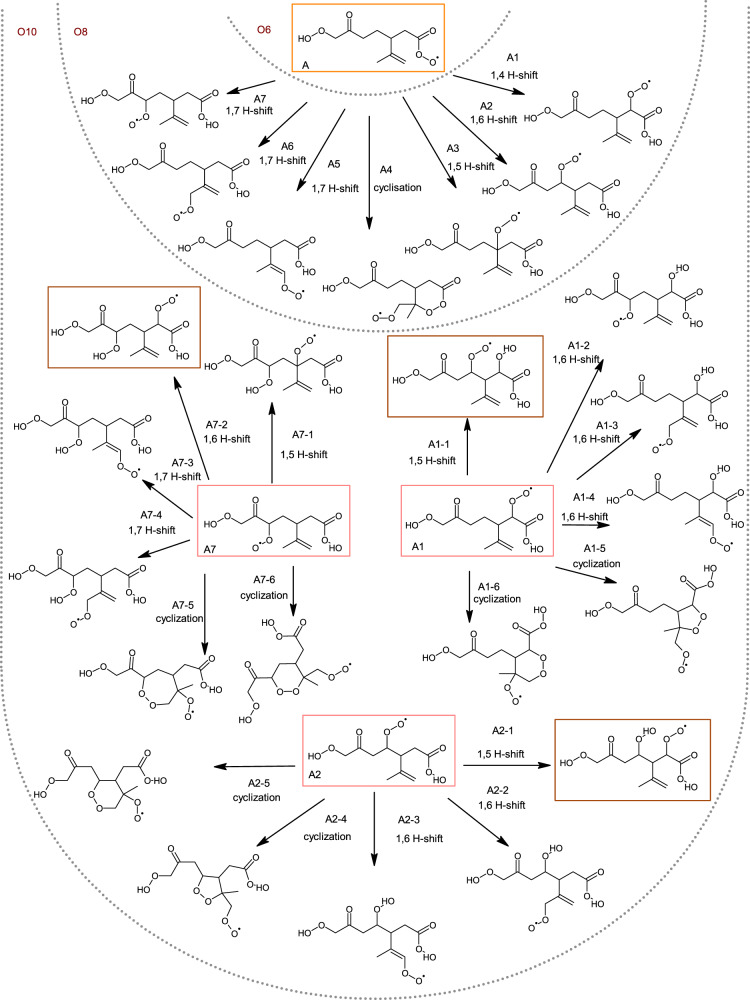
Proposed limonene autooxidation pathways and products. Potential structures and proposed formation mechanisms of the O_6_, O_8_, and O_10_ RO_2_ radicals from the A route of the ozonolysis of limonene. Most plausible identified structures are boxed in orange, pink, or brown for O_6_, O_8_, and O_10_ RO_2_ radicals, respectively.

An overview of limonene ozonolysis mechanism can be found in Fig. 2 and Supplementary Fig. 5. Briefly, limonene ozonolysis predominantly occurs by ozone addition onto the endocyclic double bond, forming an unstable primary ozonide, followed by the formation of two distinct Criegee intermediates^46–49^. The major Criegee intermediate can evolve into RO_2_ radicals, C_10_H_15_O_4_, by two main channels: A and B (Supplementary Fig. 5), which can undergo further autoxidation via intramolecular H-shifts followed by O_2_ addition, forming more oxidized RO_2_ radicals (i.e., C_10_H_15_O_6,8,10_)^17,47,48^. Earlier studies have showed that aldehydic H-shift is rapid enough (i.e., 10^−2^ to 10 s^−1^) to be competitive against bimolecular reactions^50–53^. Thus, for limonene, the aldehydic H-shift is anticipated to be a major formation pathway for C_10_H_15_O_6_ via a 1,9- or a 1,7 H-shift for A and B routes, respectively (Supplementary Fig. 5). We note that while the formation of A/B 4 via endocyclization is also expected to be fast^21,54^, the corresponding fragmentation patterns were not observed in our MS/MS spectra. Figure 2 and Supplementary Fig. 5 labels possible C_10_H_15_O_8_ radicals from both routes as A1–A7 and B1–B7, respectively. It is possible that some RO_2_ isomers may exist to varying extents. Nonetheless, offline ultra-performance liquid chromatography–electrospray ionization–tandem mass spectrometry (UPLC–ESI–MS/MS) analyses show that a limited number of isomers dominate the RO_2_ termination products (Supplementary Fig. 6), consistent with previous studies using ion mobility MS^55^. These prior studies suggest that a limited number of isomers are formed from the oxidation of monoterpenes. With this in mind, we demonstrate how MS/MS analyses can be utilized to constrain the most probable isomers and the corresponding H-shift pathways given the observed product ions, neutral losses, and known fragmentation mechanisms.

**Fig. 3:**
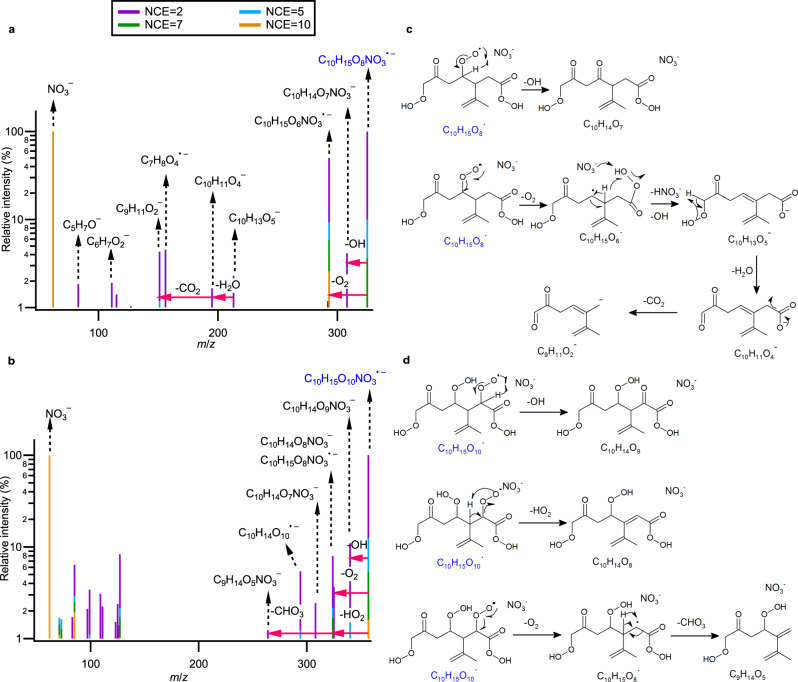
MS/MS spectra of two limonene ozonolysis RO_2_ radicals at various NCE (2–10). **a** MS/MS spectra of C_10_H_15_O_8_NO_3_^•–^. **b** MS/MS spectra of C_10_H_15_O_10_NO_3_^•–^. **c** MS/MS fragmentation routes to product ions, C_10_H_14_O_7_NO_3_^–^ and C_9_H_11_O_2_^–^ from the A2 structure of C_10_H_15_O_8_NO_3_^•–^ as depicted in Fig. 2. **d** MS/MS fragmentation route to product ions, C_10_H_14_O_9_NO_3_^–^, C_10_H_14_O_8_NO_3_^–^, and C_9_H_14_O_5_NO_3_^–^, from the structure B2-1 of C_10_H_15_O_10_NO_3_^•–^ as depicted in Supplementary Fig. 7. The parent radical anions are highlighted in blue.

Figure 3a shows that the radical C_10_H_15_O_8_NO_3_^•–^ (*m/z* 325.0644) first undergoes O_2_ elimination during fragmentation, forming the alkyl radical-anion C_10_H_15_O_6_NO_3_^•–^ (*m/z* 293.0748), followed by the simultaneous loss of HNO_3_ and OH, yielding C_10_H_13_O_5_^–^ (*m/z* 213.0766) (Fig. 3c). This can only occur if the carbon adjacent to the alkyl radical is not quaternary, therefore excluding structures A/B 4, 5, 6 and B7. The H_2_O elimination from the resulting C_10_H_13_O_5_^–^ to form C_10_H_11_O_4_^–^ (*m/z* 195.0667) likely occurs on the hydroperoxide functional group (Fig. 3c), a known negative ion fragmentation pathway^56^. The C_9_H_11_O_2_^–^ (*m/z* 151.0764) product ion results from C_10_H_11_O_4_^–^ via a charge migration elimination of CO_2,_ which is indicative of a carboxylic functional group, related to a peroxy acid moiety formed from the aldehydic H-shift. Finally, the formation of C_10_H_14_O_7_NO_3_^–^ (*m/z* 308.0616) from C_10_H_15_O_8_NO_3_^•–^ through an intramolecular H-abstraction from the peroxy radical moiety, followed by OH elimination and ketone formation, is not possible for tertiary alkylperoxy radicals such as A/B 3. Based on the MS/MS analysis, A/B 1 and 2 and A7 from 1,4, 1,6, or 1,7 H-shift emerge as the most plausible structures for C_10_H_15_O_8_, under the assumption that the A/B O_6_ peroxy radical structures were the precursors of the O_8_ radicals. Additionally, if we consider rapid H-shift scrambling (scrb)^57^, B1-3 and 7-scrb structures (with B7-scrb corresponding to A7) may also be plausible structures for C_10_H_15_O_8_, as these structures can accommodate the observed MS/MS fragmentation pathways.

Assuming A/B 1,2 and A7 as the main structures of C_10_H_15_O_8_, several C_10_H_15_O_10_ structures are possible (Fig. 1 and Supplementary Figs. 7 and 8). In the MS/MS spectrum of C_10_H_15_O_10_NO_3_^•–^ (*m/z* 357.0543, Fig. 3b), the product ion C_10_H_14_O_9_NO_3_^–^ (*m/z* 340.0515) is likely formed via OH elimination similar to that depicted in Fig. 3d, which is not possible for tertiary alkyl peroxy radicals, thereby eliminating several potential candidates (A1–6, A2–5, A7–1, A7–5, B1–6, and B2–6). The formation of C_10_H_14_O_8_NO_3_^–^ (*m/z* 324.0565) from C_10_H_15_O_10_NO_3_^•–^ via HO_2_ elimination occurs if the carbon next to the RO_2_ radical is not quaternary, limiting the potential precursor structures to A/B 1–1, A/B 1–2, A/B 2–1, and A 7–2. Following O_2_ elimination from C_10_H_15_O_10_NO_3_^•–^, the subsequent CHO_3_ elimination from C_10_H_15_O_8_NO_3_^•–^ to form C_9_H_14_O_5_NO_3_^–^ (*m/z* 264.0725) can follow a radical recombination mechanism described in Fig. 3d. Such recombination is possible if the radical is near a peroxy functional group, excluding the A/B 1–2 structures. Therefore, A/B 1–1, 2–1, and A 7–2 are likely the only candidates for C_10_H_15_O_10_, corresponding to a 1,5 and 1,6 H-shift from saturated carbon atoms, for which all observed loss processes are feasible. Lastly, we note that if H-scrambling between an RO_2_ site and hydroperoxide functional groups occurs in C_10_H_15_O_8_ and C_10_H_15_O_10_ radicals, up to 14 additional potential structures may also explain the observed O_10_ MS/MS fragmentation pattern.

As previously described and discussed in the Supplementary Information (Supplementary Figs. 9–11), the MS/MS spectrum of C_10_H_15_O_8_ from α-pinene ozonolysis (Supplementary Fig. 10a) exhibits similar fragmentations (i.e., O_2_ and CO_2_ eliminations corresponding to the RO_2_ radical moiety and the peroxy acid functional group, respectively) but also some notable differences compared to products from limonene oxidation. Previous works investigated the fragmentation of compounds bearing a peroxy acid functional group and described a common fragmentation loss via CH_2_O_2_ elimination through a two-step fragmentation process (H_2_O + CO)^58,59^, which is not observed for either α-pinene or limonene. In addition, the MS/MS spectrum of α-pinene C_10_H_15_O_8_^–^ RO_2_ radical displays two distinct fragmentation losses, CH_3_O_2_ and C_3_H_6_O, which are indicative of the methyl group specific to α-pinene RO_2_, i.e., quaternary carbon bonded to two methyl groups (Supplementary Fig. 9, highlighted in blue), which is not present in the case of limonene RO_2_.

### Gas-phase dimer formation mechanism

**Fig. 4:**
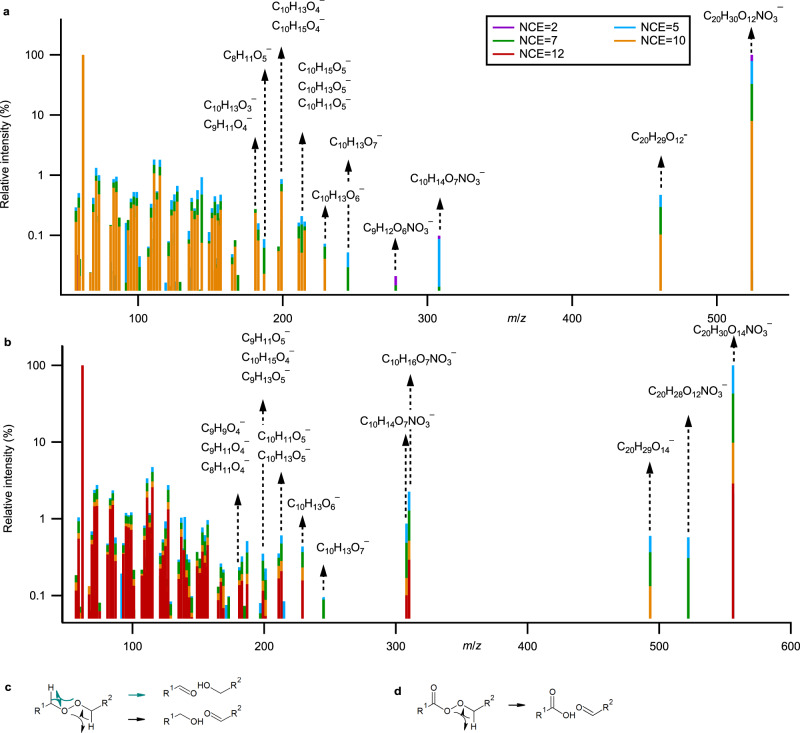
MS/MS spectra of two limonene ozonolysis dimers at various NCE (2–10). **a** MS/MS spectra of C_20_H_30_O_12_NO_3_^–^. **b** MS/MS spectra of C_20_H_30_O_14_NO_3_^–^. **c** MS/MS fragmentation mechanism of RO–OR for dimers produced from the reaction involving two peroxy radical precursors, which can proceed via either of the black or green pathways. **d** MS/MS fragmentation mechanism of RO–OR for dimers produced from the reaction involving an acylperoxy radical and a peroxy radical, where only one pathway is available.

The reaction rate coefficient between RO_2_ radicals can range from 10^−17^ to 10^−11^ cm^3^ molecule^−1^ s^−1^ ^60^, with recent studies reporting values on the order of 10^−10^ cm^3^ molecule^−1^ s^−1^ for selected HOM-RO_2_ ^21,61^. While kinetic studies show that the RO_2_ + RO_2_ → ROOR + O_2_ reaction rate coefficients generally increase with the RO_2_ oxygen content^62^, further enhanced by intramolecular hydrogen bonds that may help with transition state stabilization^28^, the contribution of different RO_2_ radicals to dimer formation remains unknown. Online MS/MS analysis provides insights into the dimer structure and formation, as shown for C_20_H_30_O_12_NO_3_^–^ (*m/z* 524.1608) and C_20_H_30_O_14_NO_3_^–^ (*m/z* 556.1520) in Fig. 4 for limonene ozonolysis and in Supplementary Fig. 12 for α-pinene ozonolysis. C_20_H_30_O_12_ can be formed from reactions of O_4_ with O_10_ or O_6_ with O_8_ peroxy radicals; C_20_H_30_O_14_ can be formed from reactions of O_8_ with O_8_, or O_6_ with O_10_ peroxy radicals. As shown in Fig. 4a and b, both limonene ozonolysis dimers produce the C_10_H_14_O_7_NO_3_^–^ (*m/z* 308.0617) product ion, which is likely formed from the cleavage of the peroxide (i.e., RO–OR bond) into an alkoxy radical (RO) combined with H-exchange, as illustrated in Fig. 4c. The presence of C_10_H_14_O_7_NO_3_^–^ suggests that C_10_H_15_O_8_ may be the main precursor for C_20_H_30_O_12_ and C_20_H_30_O_14_. This is also supported by the absence of O_9_ fragments and the presence of C_10_H_13_O_7_^–^ (*m/z* 245.0663). This is further reinforced by hierarchical clustering analyses (Supplementary Fig. 13), which consistently group C_10_H_16_O_8_ (termination product of C_10_H_15_O_8_), C_20_H_30_O_12_, and C_20_H_30_O_14_ together based on their MS/MS spectral similarity. The hierarchical clustering analysis also reveals that the MS/MS spectrum of C_10_H_16_O_7_NO_3_^–^ is distinct from that of C_20_H_30_O_12_NO_3_^–^ and C_20_H_30_O_14_NO_3_^–^ (Supplementary Fig. 13). Similarly, the importance of the O_6_-radical (i.e., C_10_H_15_O_6_) in the formation of the C_20_H_30_O_12_ is supported by the detection of C_10_H_15_O_5_^–^ (*m/z* 215.0921). While C_20_H_30_O_14_NO_3_^–^ produces a C_10_H_16_O_7_NO_3_^–^ (*m/z* 310.0773) product ion (Fig. 4b), C_20_H_30_O_12_NO_3_^–^ does not (Fig. 4a). This difference indicates that C_10_H_15_O_6_ is an acylperoxy radical, which is consistent with the structure proposed in Fig. 4d. As a result, only one RO–OR cleavage pathway for C_20_H_30_O_12_NO_3_^–^ is possible with the H-exchange only taking place in one side of the peroxide, in contrast to C_20_H_30_O_14_NO_3_^–^ (Fig. 4c). This result also supports the structures A/B proposed in Fig. 2 and Supplementary Fig. 5.

The fragmentation patterns of α-pinene dimers are distinct from those of limonene, suggesting a considerable difference in the reactivity of their RO_2_ radicals (as discussed below). For example, the reaction between C_10_H_15_O_10_ and C_10_H_15_O_4_ RO_2_ radicals clearly contributes to the formation of C_20_H_30_O_12_ dimer from α-pinene ozonolysis, according to the presence of C_10_H_16_O_9_NO_3_^–^ (*m/z* 342.0676) and C_10_H_15_O_9_^–^ (*m/z* 279.0722) product ions in the MS/MS spectrum, as shown in Supplementary Fig. 12. The C_10_H_15_O_6_^–^ (*m/z* 231.0874) product ion observed in the MS/MS spectrum might suggest that RO_2_ radicals other than O_4_ and O_10_ may contribute to the formation of dimer, although this product ion may have also originated from further fragmentation of a larger product ion. Hence, reactions between C_10_H_15_O_6_ and C_10_H_15_O_8_ radicals may also contribute to the formation of C_20_H_30_O_12_ dimers, though the corresponding RO–OR product ions, e.g., C_10_H_16_O_5_NO_3_^–^ and C_10_H_16_O_7_NO_3_^–^, were not observed under our experimental conditions. The greater importance of the O_4_ α-pinene RO_2_ radical compared to the O_4_ limonene RO_2_ radical is also reflected in the dimer concentration distributions (Supplementary Fig. 1d), with less oxygenated dimers being more abundant during α-pinene ozonolysis (C_20_H_30_O_10_ and C_20_H_30_O_12_) than during limonene ozonolysis (C_20_H_30_O_12_ and C_20_H_30_O_14_). Ozonolysis experiment with varying α-pinene concentration further indicates that the O_4_ α-pinene RO_2_ radical is a key participant in the formation of C_20_H_30_O_8_ (with C_10_H_15_O_6_), C_20_H_30_O_10_ (with C_10_H_15_O_8_), and C_20_H_30_O_12_ (with C_10_H_15_O_10_) dimers (see Supplementary Figs. 15, 16 and Supplementary Information).

## Discussion

We have shown that the structures A/B-1, A/B-2, and A-7 may be potential RO_2_ radicals formed during limonene autoxidation. The formation of these structures requires 1,6-, 1,7-, and 1,4-H shifts, respectively. For A1 and B1, the required 1,4 H-shifts are typically thought to be slow, due to steric hinderance^10^, but theoretical calculations show that 1,4-H-aldehydic migration has significantly lower energy barriers than other H-shift pathways on unsubstituted aliphatic carbons^63^. The presence of adjacent functional groups such as alkyl, carbonyls, hydroxyl, hydroperoxyl, and ether can further enhance H-abstraction^10,64^. Unlike 1,4 H-shifts, 1,6 and 1,7 H-shifts are not expected to cause steric strains, though H-abstraction from a saturated carbon atom is likely slow^63^. Structures resulting from a saturated carbon H-shift were shown to be good candidates and could explain MS/MS fragmentation for both limonene and α-pinene peroxy radicals in our work, despite their structural differences. Our data also highlight the presence of peroxy acid functional groups for both limonene and α-pinene peroxy radicals, which has also been suggested for other VOCs^16,65^. This is further supported by offline UHPLC–ESI–MS/MS analyses of C_10_H_16_O_7–8_ and C_10_H_16_O_5–6_ (termination products of the O_8_ and O_6_ peroxy radicals, respectively), largely detected in the negative ion mode (Supplementary Fig. 6), which is consistent with the presence of an acidic hydrogen from the peroxy acid functional group ^58,59^.

When applied to gaseous dimers, online MS/MS reveals unique RO–OR dimer fragmentation pathways that help identifying main RO_2_ radical precursors, which is further supported by hierarchical clustering analyses. Our results show that HOM dimer formation during limonene ozonolysis is driven by the O_8_ peroxy radical (Fig. 4 and Supplementary Figs. 14 and 16), whereas the O_4_ peroxy radical appears to be a key component for dimer formation during α-pinene ozonolysis (Supplementary Figs. 12, 14, and 16). Consequently, dimers produced from α-pinene ozonolysis were on averaged less oxidized than those produced from limonene ozonolysis (Supplementary Fig. 1d) despite seemingly similar RO_2_ radical distribution (O_8_ > O_10_ > O_6_, Supplementary Fig. 1c), with potential implications of reduced NPF due to the higher vapor pressure of α-pinene dimers^22^. Furthermore, our results show that the RO_2_ + RO_2_ reaction rate coefficient is not only driven by the degree of oxidation^61^ (Supplementary Fig. 16) but also by the structure/reactivity of each RO_2_ radicals. Overall, our findings advance the understanding of atmospheric radical chemistry, which can help constraining model representation of autoxidation pathways and dimer formation kinetics. The online approach employed here can be readily applied to, and is beneficial for, the investigation of short-lived or labile organic compounds in the gas phase present in low concentrations, demonstrably organic radicals that are otherwise inaccessible by offline techniques.

## Methods

### General information and procedures

Ozonolysis experiments were performed in a 18-liter Pyrex glass flow tube reactor (12 cm i.d. × 158 cm length) at room temperature^66^. Continuous injection of volatile organic compound (VOC) precursors, (+)-limonene (Sigma-Aldrich, 97% purity) or (+)-α-pinene (Sigma-Aldrich, ≥99%), ozone, and flow reactor carrier gas was regulated using mass flow controllers (MFC, Bronkhorst). Dry synthetic air (80:20 N_2_:O_2_) was used as the carrier gas for the flow tube and VOC injection. Total flow rate is kept at 21 L min^−1^ and the total reaction time is 59.1 s. Input VOC concentrations, ranging from 45 to 227 ppbv for limonene and 214 to 749 ppbv for α-pinene, are estimated based on MFC settings and VOC evaporator temperature (5 °C). Ozone was generated via dielectric barrier discharge and was monitored with a Thermo 49C analyzer, ranging from 20 to 40 ppbv. Negligible particle formation (<100 cm^−3^) was observed by a condensation particle counter (TSI CPC 3772) during all experiments. A Q Exactive Orbitrap mass spectrometer (Thermo Scientific, US) coupled to an atmospheric pressure CI inlet^44^ was used for online analysis of ozonolysis products. The Orbitrap has been used with an automatic gain control (AGC) and maximum injection time set to 1 × 10^5^ and 3000 ms, respectively. The mass resolution is set to 140,000 at *m/*z 200. The CI reagent nitrate ion (NO_3_^–^) was generated from a nitric acid solution (Sigma-Aldrich, 65% purity) continuously flushed with pure N_2_ (10 mL min^−1^) and ionized with a soft X-ray photoionizer (Hamamatsu, L9491). The CI inlet total flow and sheath flow rates were set at 36 L min^−1^ and 34 L min^−1^, respectively. Further details of instrument setting and experimental conditions can be found in the Supplementary information.

### Online tandem MS

Higher energy collision dissociation (HCD) was used to obtain targeted MS/MS spectra using a quadrupole ion isolation window width of 0.4 Da. No in-source collision-induced dissociation (CID) energy was applied. The HCD cell is located after the quadrupole capable of mass isolation within ±0.4 Da. The selectivity of the NO_3_^–^ detection scheme toward acidic and highly oxidized compounds ensures that a single parent ion is selected for structural characterization using MS/MS acquisitions for the RO_2_ radicals and dimeric products discussed in the main text within the mass isolation window. In the case that more than one parent ion is isolated, the dominant parent ion is at least an order of magnitude more abundant than the other parent ion(s) in most cases. An NCE of 2 was applied to RO_2_ and closed-shell monomers. An NCE value of 5 was applied to dimers, as well as to select RO_2_ and monomers for comparison. Systematic ramping of NCE from 2–10 was also performed for a few targeted compounds. The Orbitrap was mass-calibrated using an aqueous sodium acetate solution (2 mM, Aldrich, >99% purity). During online CI-measurements, the drift in mass accuracy was negligible (i.e., <2 p.p.m.) based on the reported *m/z* of the CI reagent ions (NO_3_^–^ and HNO_3_NO_3_^–^) and *m/z* values from MS/MS parent ions, which is within the specifications of the Orbitrap. Peak identification and assignment were performed using XCalibur 4.1 (Thermo Scientific). Identification of parent ion formula was constrained based on known oxidation chemistry and elemental composition, i.e., ions are assumed to contain only carbon, hydrogen, and oxygen atoms with up to two nitrogen atoms to account for adduction with NO_3_^–^ or HNO_3_NO_3_^–^. Identification of MS/MS product ion is constrained by the parent ion elemental composition. Product ion intensities were normalized to the maximum ion intensity unless stated otherwise. A 5 p.p.m. *m/z* tolerance and a 0.1% relative intensity threshold were applied to refine the product ion lists. Agglomerative hierarchical clustering of the processed MS/MS spectra was performed in Spyder 3.3.2 using complete linkage of cosine distance of square root-transformed product ion relative intensities as recommended in the literature^67^, excluding NO_3_^–^ product ions, whose relative contribution reflects more the dissociation energy of NO_3_^–^ adducts than the analyte structure or functionalization. The precursor ion signal is the most intense in all MS/MS spectra acquired at NCE ≤ 5 after excluding the NO_3_^–^ ion. Clustering analysis results based on observed product ions alone or together with inferred neutral losses are detailed in the Supplementary Information. Details of the offline analysis of monoterpene ozonolysis products using high-performance liquid chromatography with Orbitrap are found in the Supplementary Information.

## Supplementary information

Supplementary Information

Peer Review File

## Data Availability

The data supporting the finding of this study are available in this article and Supplementary information. Data presented in the main text are accessible via 10.5281/zenodo.4276954. Raw mass spectrometric data are available from the corresponding author on reasonable request.
