# Supplementary material for: Structures and reactivity of peroxy radicals and dimeric products revealed by online tandem mass spectrometry
Source: Nat Commun. 2021 Jan 12;12:300. doi: 10.1038/s41467-020-20532-2 (PMC7804243; doi:10.1038/s41467-020-20532-2)
Supplement: Supplementary file 1 — Supplementary Information [file 41467_2020_20532_MOESM1_ESM.pdf]

Structures and Reactivity of Peroxy Radicals and Dimeric Products Revealed by Online Tandem Mass Spectrometry

Sophie Tomaz[1,*], Dongyu Wang[2,*], Nicolás Zabalegui[3,4], Dandan Li[1], Houssni Lamkaddam[2], Franziska Bachmeier[5], Alexander Vogel[5], María Eugenia Monge[3], Sébastien Perrier[1], Urs Baltensperger[2], Christian George[1], Matti Rissanen[6,7], Mikael Ehn[6], Imad El-Haddad[2], Matthieu Riva[1,†]

[1] Univ Lyon, Université Claude Bernard Lyon 1, CNRS, IRCELYON, 69626, Villeurbanne, France
[2] Laboratory of Atmospheric Chemistry, Paul Scherrer Institute, 5232 Villigen, Switzerland
[3] Centro de Investigaciones en Bionanociencias (CIBION), Consejo Nacional de Investigaciones Científicas y Técnicas (CONICET), Godoy Cruz 2390, Ciudad de Buenos Aires, C1425FQD, Argentina
[4] Departamento de Química Inorgánica Analítica y Química Física, Facultad de Ciencias Exactas y Naturales, Universidad de Buenos Aires, Ciudad Universitaria, Buenos Aires, C1428EGA, Argentina
[5] Institute for Atmospheric and Environmental Sciences, Goethe-University Frankfurt, 60438 Frankfurt am Main, Germany
[6] Institute for Atmospheric and Earth System Research, INAR /Physics, Faculty of Science, University of Helsinki, Helsinki, FI–00014, Finland
[7]Aerosol Physics Laboratory, Physics Unit, Faculty of Engineering and Natural Sciences, Tampere University, FI-33101, Tampere, Finland.
*These authors contributed equally to this work

†Corresponding author: Matthieu Riva – email : matthieu.riva@ircelyon.univ-lyon1.fr

Supplementary Figures

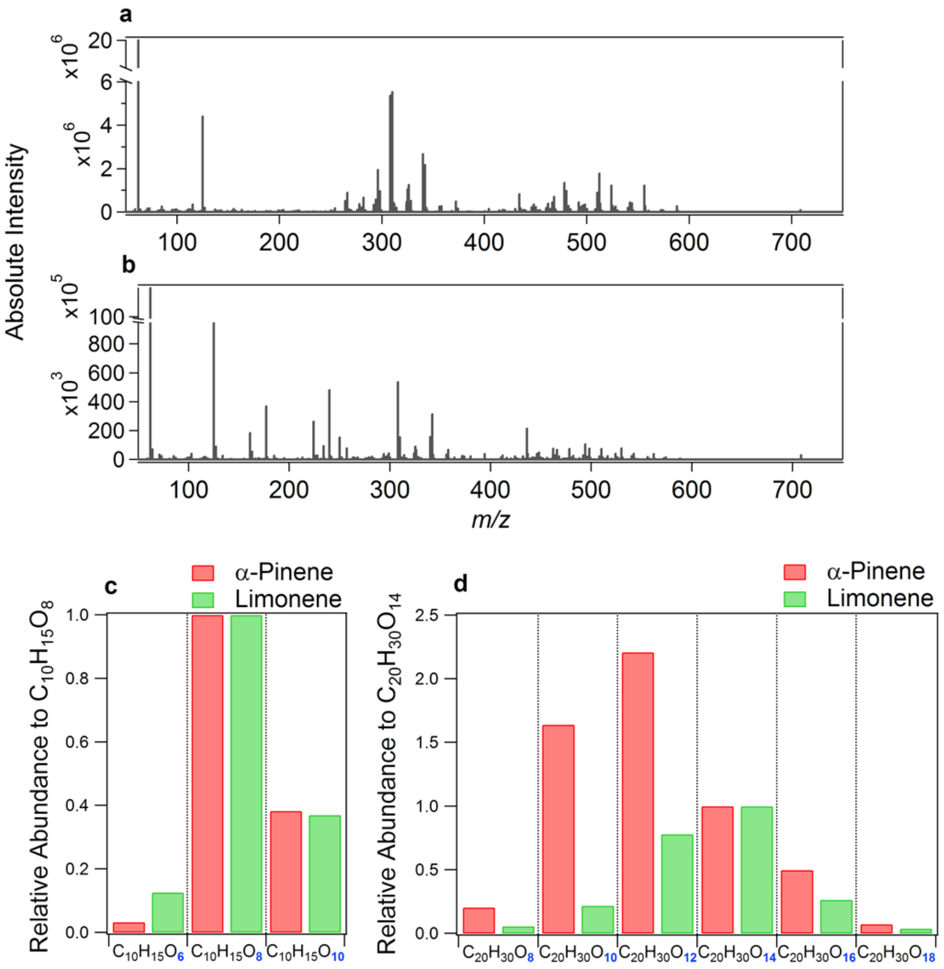

Supplementary Figure 1. MS spectra of HOMs generated from the O_3-initiated oxidation of **(a)** limonene, or **(b)** α-pinene, and the corresponding **(c)** abundance of $C_{10}H_{15}O_{8\text{-}12}$ radicals relative to that of $C_{10}H_{15}O_8$, and **(d)** the abundance of $C_{20}H_{30}O_{8\text{-}18}$ dimers relative to that of $C_{20}H_{30}O_{14}$. MS spectra shown each represent a 5-minute average of online MS acquired at steady-state.

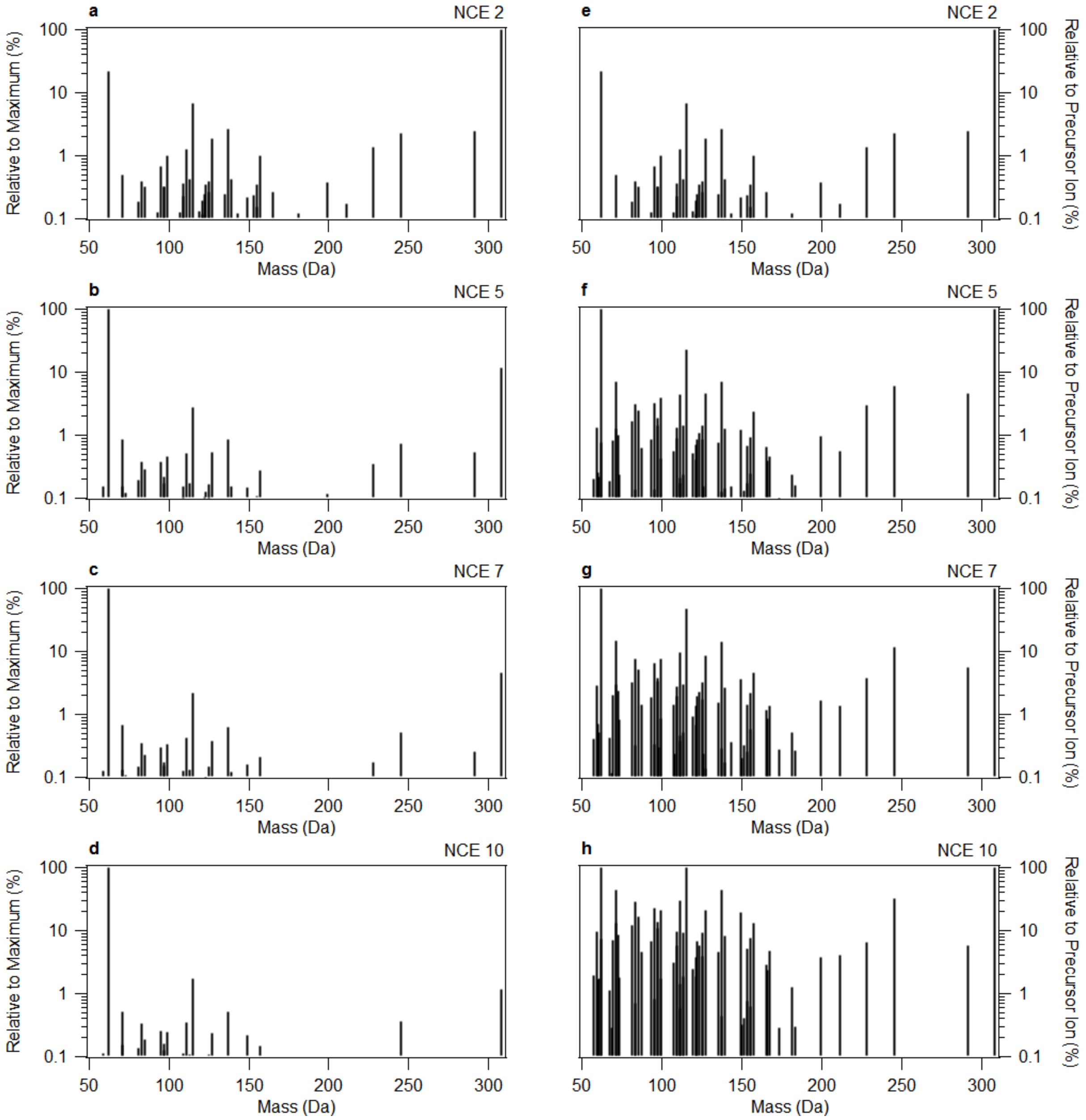

Supplementary Figure 2. MS/MS spectra of $C_{10}H_{14}O_7\ NO_3^-$ precursor ion at *m/z* 308.0623 observed during limonene ozonolysis. Tandem MS spectra were acquired at NCE (**a** & **e**) 2, (**b** & **f**) 5, (**c** & **g**) 7 and (**d** & **h**) 10. Product ion signals are normalized to either the maximum ion signal intensity (a-d) or the precursor ion signal intensity (e-h). For NCE > 2, the abundance of NO_3^- (*m/z* 61.9884) exceeds that of the precursor ion. For NCE ≥ 10, the abundance of $C_5H_7O_3^-$ (*m/z* 115.0401) exceeds that of the precursor ion.

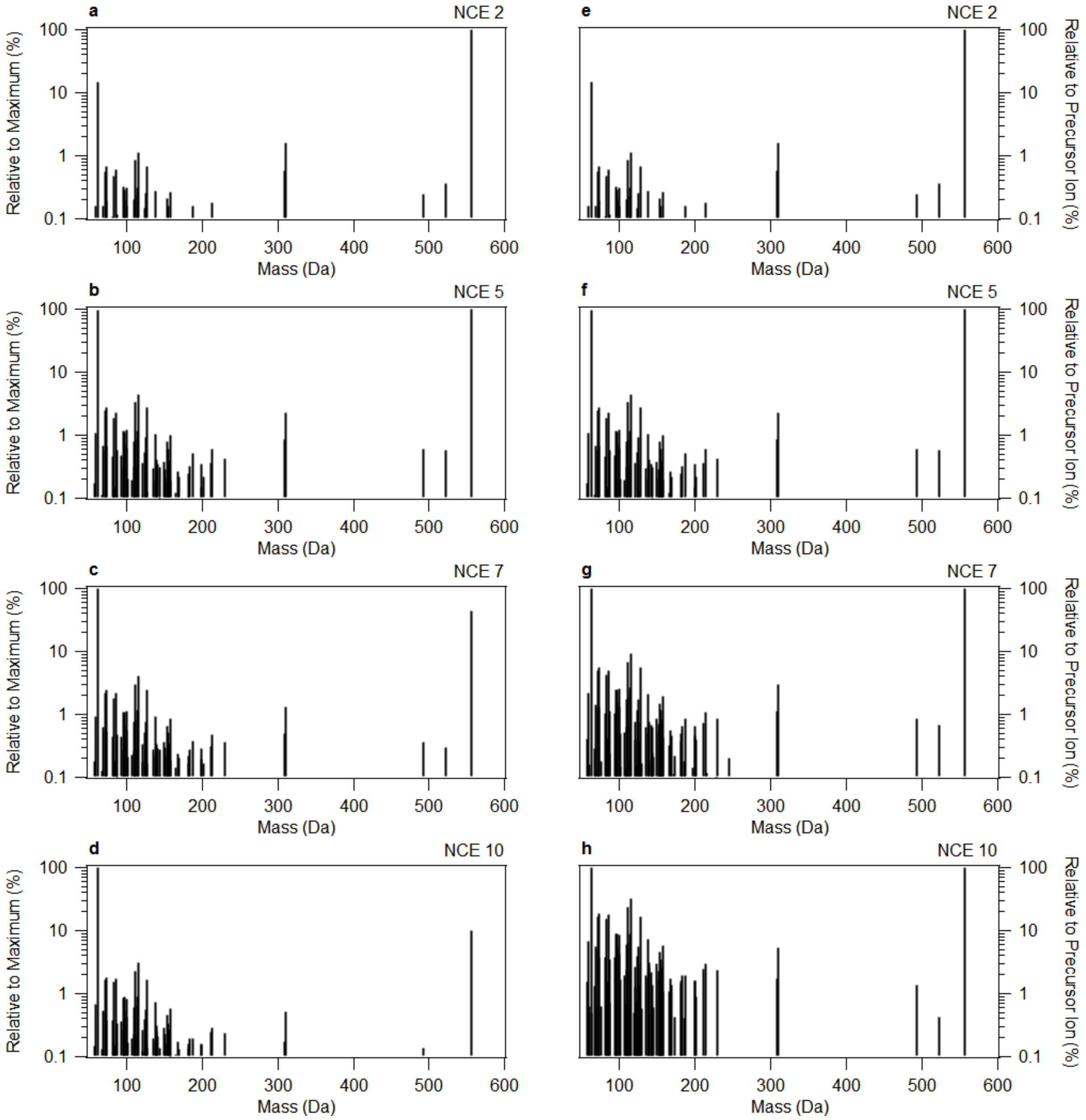

Supplementary Figure 3. MS/MS spectra of $C_{20}H_{30}O_{14}NO_3^-$ at *m/z* 556.1519 observed during limonene ozonolysis. Tandem MS spectra were acquired at NCE (**a** & **e**) 2, (**b** & **f**) 5, (**c** & **g**) 7 and (**d** & **h**) 10. Product ion signals are normalized to either the maximum ion signal intensity (**a-d**) or the precursor ion signal intensity (**e-h**). For NCE ≥ 7, the abundance of NO_3^- exceeds that of the precursor ion.

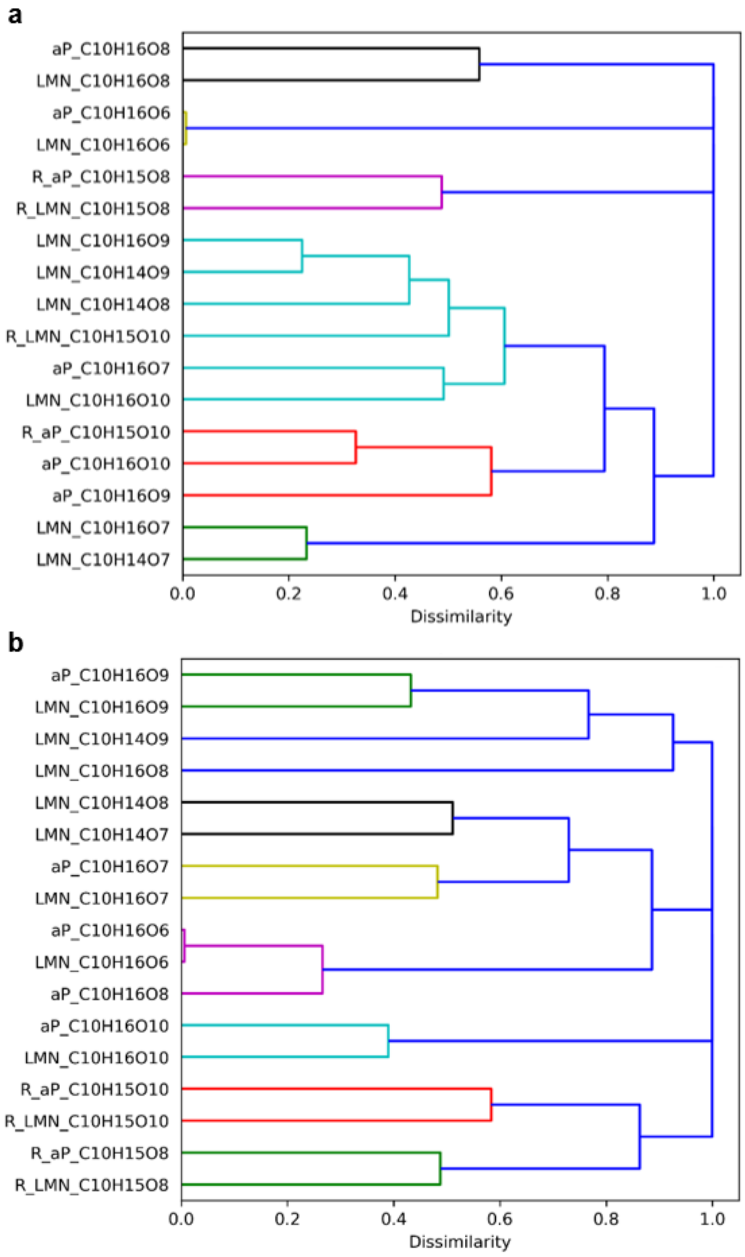

Supplementary Figure 4. Agglomerative hierarchical clustering analyses of MS/MS spectra of α-pinene and limonene ozonolysis products at NCE 2 based on (**a**) observed product ions or (**b**) neutral losses. NO_3^- as a product ion and the corresponding neutral loss from the precursor ion are excluded from the analysis. Precursor molecular formulae are shown on the y-axis, where the prefixes indicate the VOC precursor or functionality: "LMN_" for limonene, "aP_" for α-pinene, and "R_" for RO_2 radical. A similarity threshold of 0.7 was used to allocate clusters, represented by different colors. Clustering using product ions in (**a**) tend to group together compounds derived from the same VOC precursor, including both RO_2 radicals and closed-shell products. A 0 dissimilarity between aP_$C_{10}H_{16}O_6$ and LMN_$C_{10}H_{16}O_6$ is the result of their sparse MS/MS spectra, which consist of only the precursor and the NO_3^- ions. Clustering based on neutral losses in (b) was more successful for clustering of RO_2 radicals, at the cost of increased clustering of compounds derived from different VOCs, as well as the increased number of non-clustered compounds.

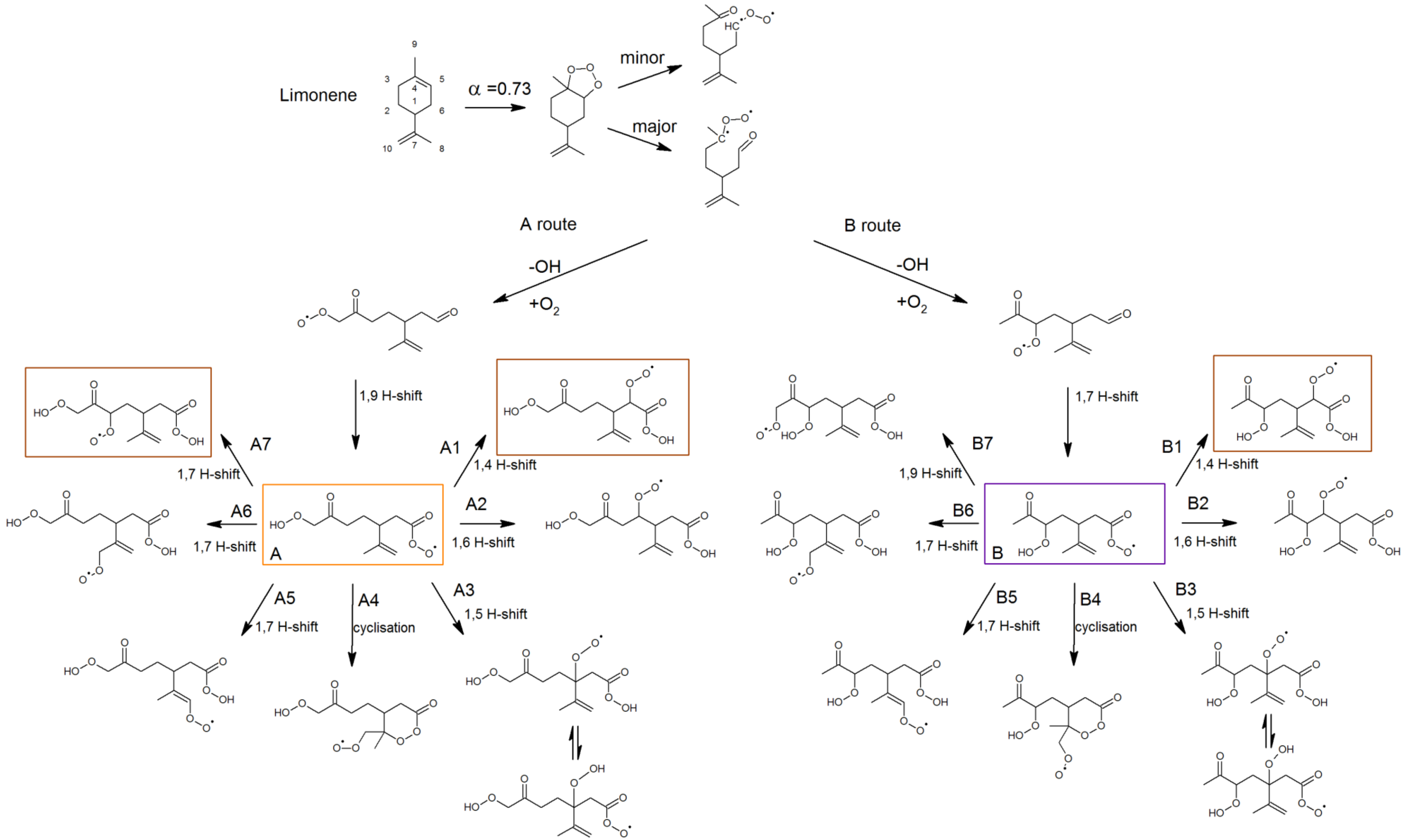

Supplementary Figure 5. Proposed formation mechanisms and potential structures for the formation of the O_8 peroxy radical from the ozonolysis of limonene. Most plausible structures are boxed in orange and purple for O_6 peroxy radicals and in brown for O_8 radicals.

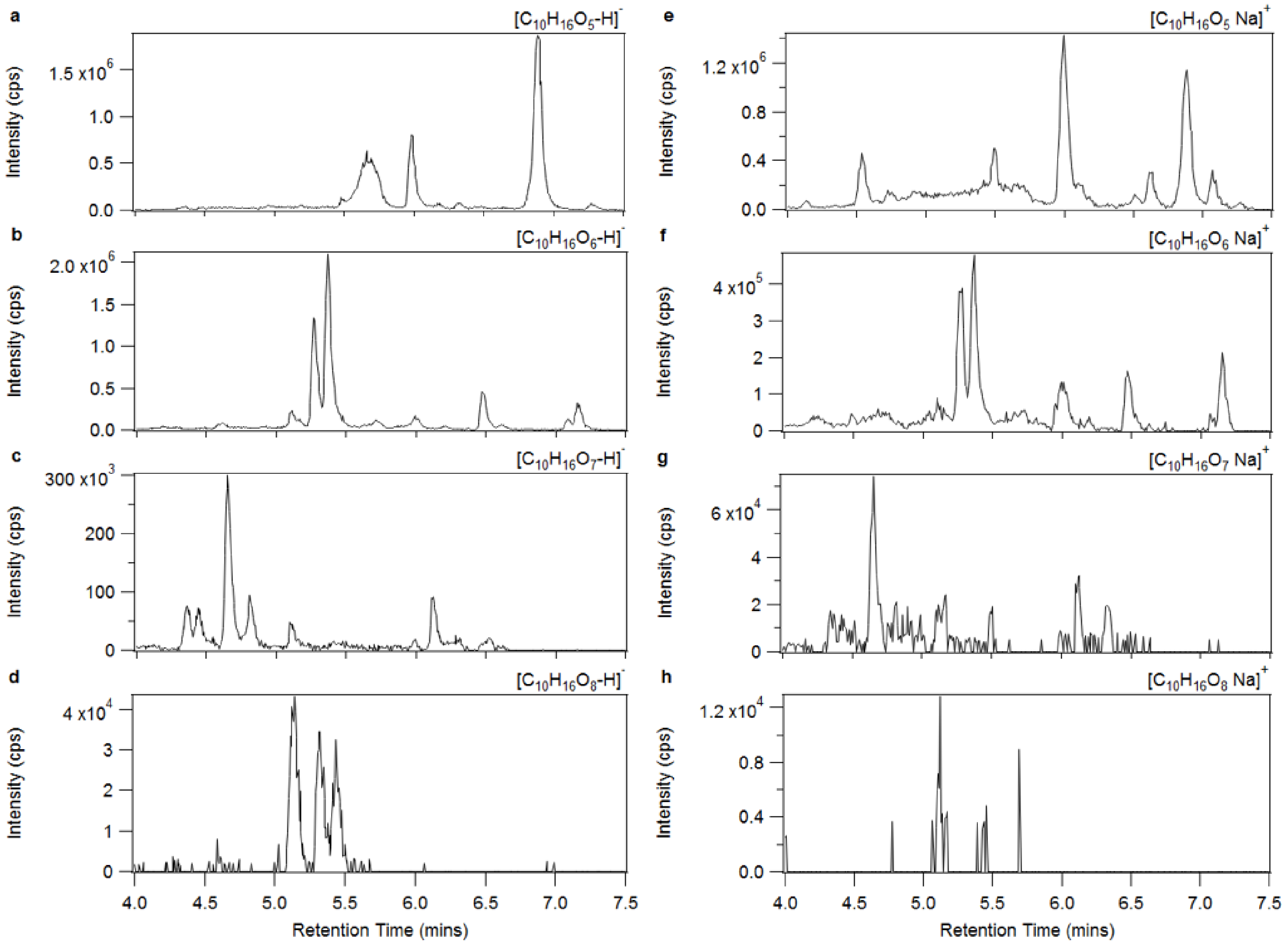

Supplementary Figure 6. Offline ultra-high-performance liquid chromatography-electrospray ionization tandem mass spectrometry (UPLC-ESI-MS/MS) chromatogram of filter extracts in (-) and (+) ionization mode.

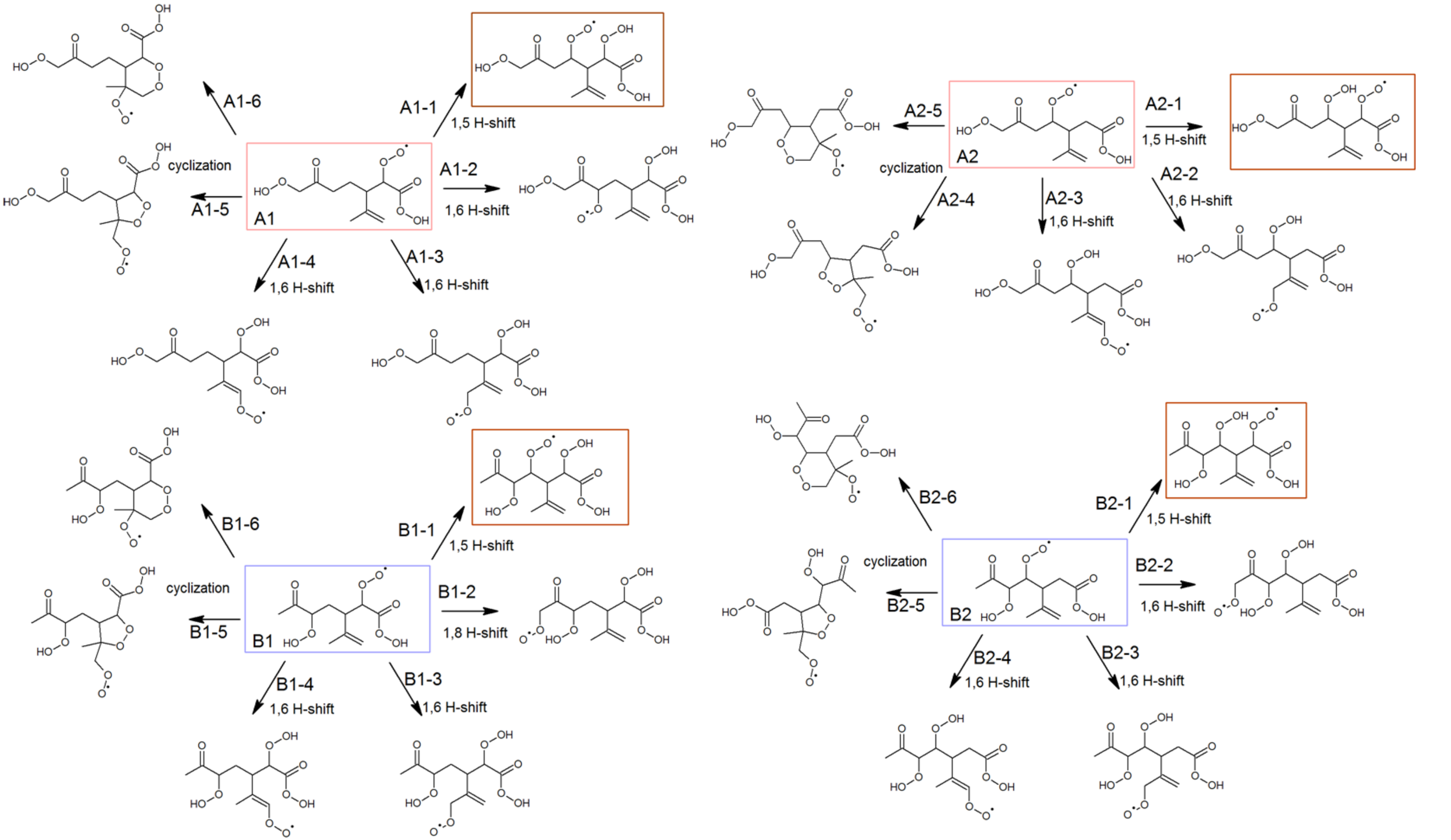

Supplementary Figure 7. Potential structures for the O_{10} peroxy radicals arising from the ozonolysis of limonene from A/B 1 and 2. Most plausible identified structures are boxed in pink and blue for O_8 radicals and in brown for O_{10} peroxy radicals.

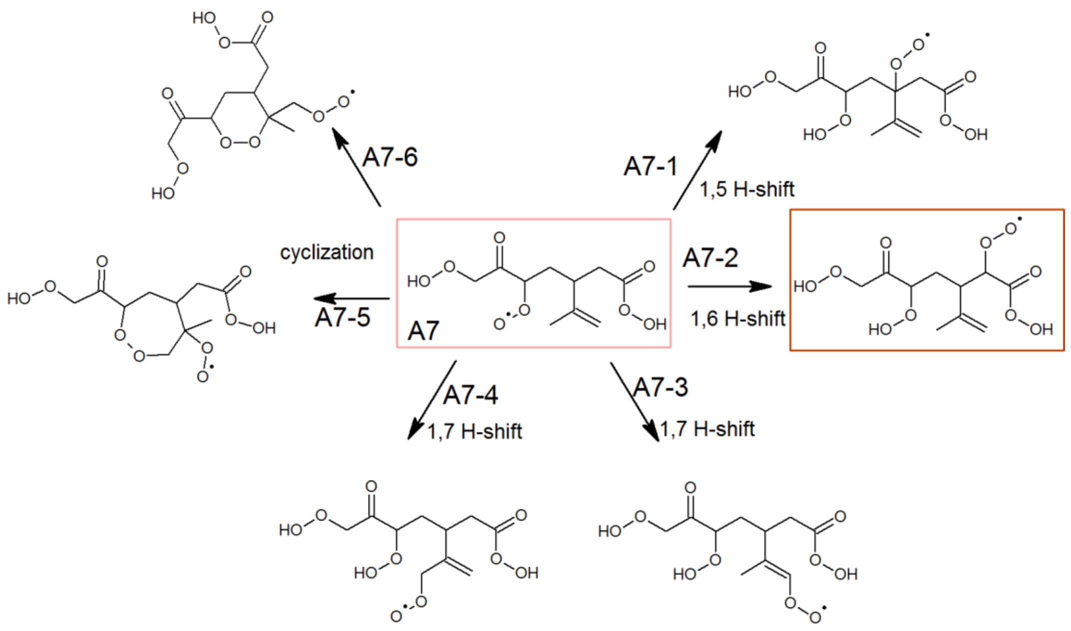

Supplementary Figure 8. Potential structures for the O_{10} peroxy radicals arising from the ozonolysis of limonene from A7. Most plausible identified structures are boxed in pink and brown for O_8 and O_{10} peroxy radicals, respectively.

Peroxy radical formation during α-pinene ozonolysis

An overview of the α-pinene ozonolysis mechanism can be found in Supplementary Figure 9. Theoretical calculations suggest that the formation of $C_{10}H_{15}O_6$ is faster via 1,4- or 1,7 shift of an aldehydic hydrogen,[1,2] as illustrated by pathway A and D, respectively. However, these calculations indicate that autoxidation would stop at $C_{10}H_{15}O_6$ since the H-abstraction from the methyl functional group or the cyclobutyl ring is expected to be very slow ($<10^{-2}$ s^{-1}) with a high energy barrier.[1] Alternatively, $C_{10}H_{15}O_6$ formation has been suggested to proceed via the opening of the cyclobutyl ring from the Criegee intermediate as illustrated by pathways E & F in Supplementary Figure 9.[1,2] The resulting $C_{10}H_{15}O_6$ is expected to undergo further autoxidation and can explain HOM formation.

The MS/MS spectrum for the α-pinene $C_{10}H_{15}O_8$ is shown in Supplementary Figure 10. The elimination of CH_3O_2 (i.e. O_2 + CH_3) from $C_{10}H_{15}O_8NO_3^{\bullet-}$ (*m/z* 325.0644) yielding $C_9H_{12}O_6NO_3^-$ (*m/z* 278.0517) is indicative of an alpha methyl group, which is only possible for A3, D4 and E6 structures. The corresponding fragmentation mechanism is described in Supplementary Figures 10b and 10c for candidates E6 and A3, respectively. The formation of $C_7H_9O_7NO_3^{\bullet-}$ (*m/z* 267.0233) can be explained by a charge remote fragmentation,[3] leading to the elimination of H_2 and C_3H_4O (Supplementary Figure 10b). Furthermore, the elimination of CO_2 from $C_7H_7O_4^-$ (*m/z* 155.0352) forming $C_6H_7O_2^-$ (*m/z* 111.0451) indicates the presence of a peroxy acid functional group. In sum, A3, D4 and E6 are likely the only candidates for $C_{10}H_{15}O_8$, for which all observed loss processes can be explained. Note that the formation of the only plausible ring-opening pathway candidate, E6, involves a 1,9-H-abstraction from a methyl group, which is likely to be slow. In addition, scrambling structures of the O_8 peroxy radicals arising from the E route would also be able to explain the observed MS/MS fragmentation.

For $C_{10}H_{15}O_{10}$, the structure may arise from A3, D4 and E6 as proposed in Supplementary Figure 10. The MS/MS spectrum (Supplementary Figure 9d) shows the elimination of $C_3H_7O_5$ and $C_2H_3O_4$ + HNO_3 leading to the formation of $C_7H_8O_5NO_3^-$ and $C_8H_{11}O_6^-$, respectively, but does not provide enough information to identify the main fragmentation pathways and identify the most probable structures even considering other potential $C_{10}H_{15}O_8$ structures. It should be noted that A3-1/5, D4-1/5 result from a primary carbon H-shift and are likely slow. The structures A3-2/3/4 and D4-2/4 involve an H-abstraction from the cyclobutyl ring and are geometrically hindered. In the case of the ring-opening structures, E6-2 formed from a double-bound H-shift is considered as unlikely while the other structures may be more plausible.

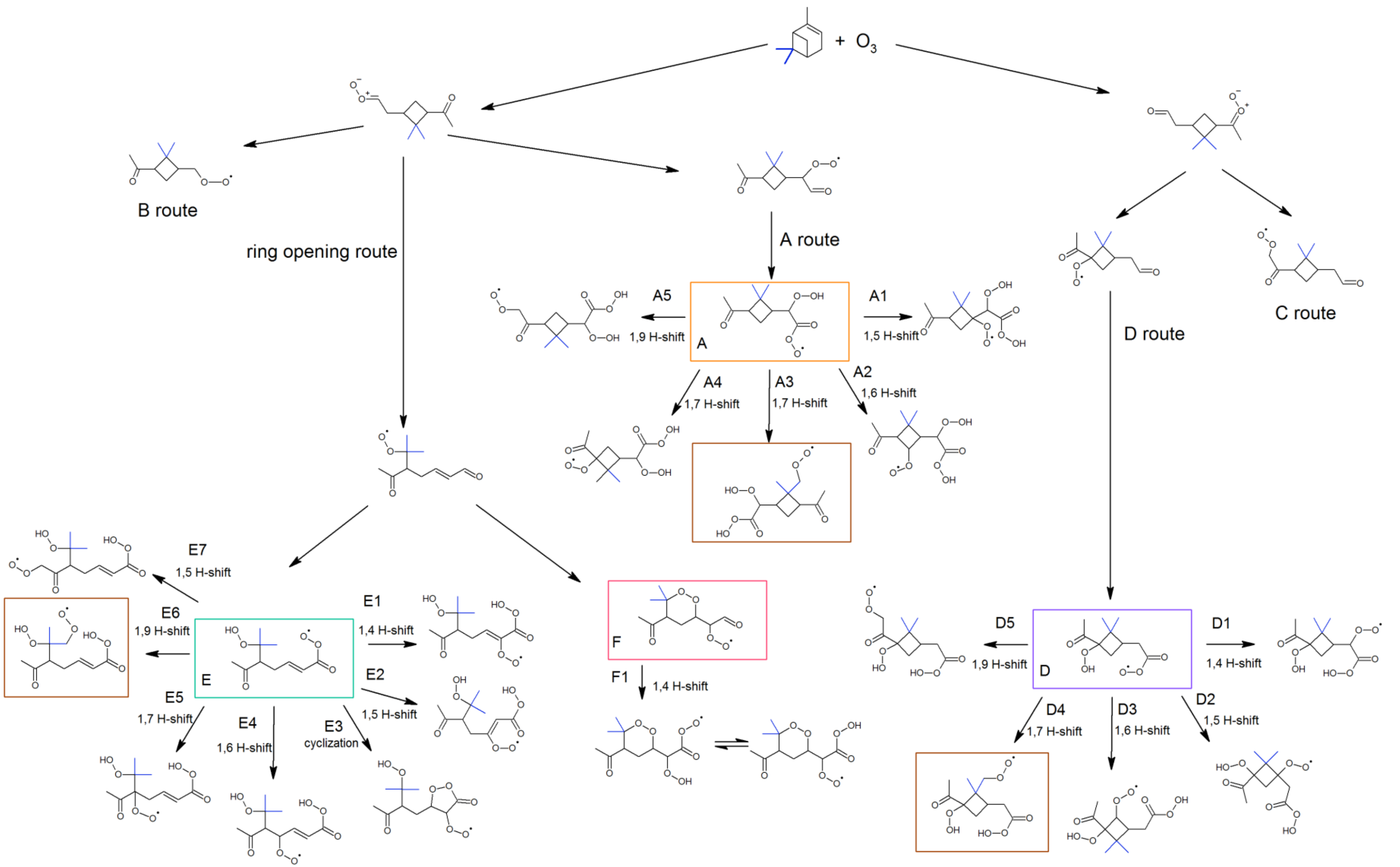

Supplementary Figure 9. Mechanism for the ozonolysis of α-pinene and potential structures for the ring-retaining and ring-opening O_8 peroxy radicals. Most plausible identified structures are boxed in orange, pink, green and purple for O_6 peroxy radicals and in brown for O_8 radicals.

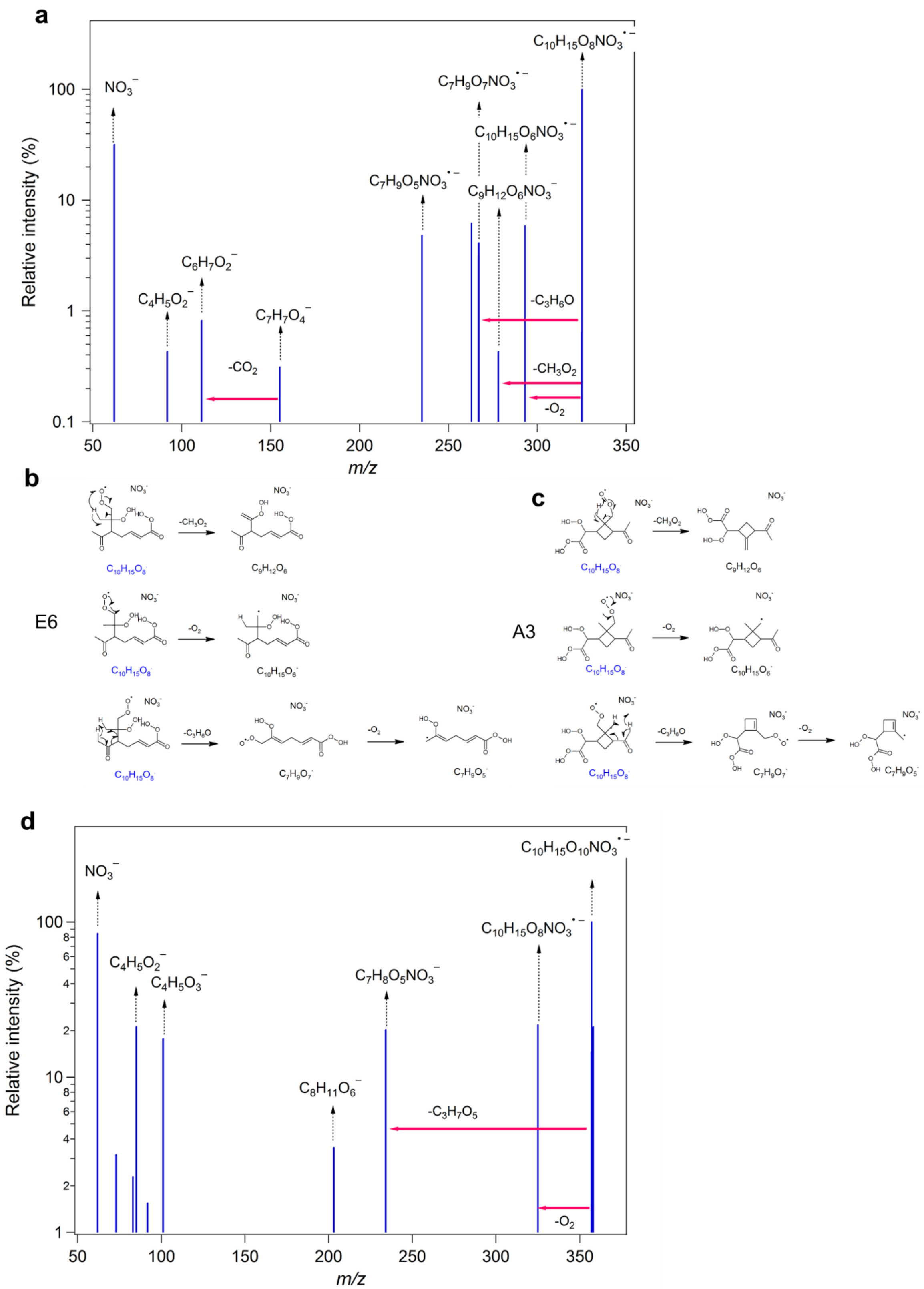

Supplementary Figure 10. MS/MS spectra at NCE=2 of **(a)** $C_{10}H_{15}O_8NO_3^{\bullet -}$ and **(d)** $C_{10}H_{15}O_{10}NO_3^{\bullet -}$ radical precursor anions formed during the ozonolysis reaction of α-pinene. Fragmentation routes of two potential $C_{10}H_{15}O_8NO_3^{\bullet -}$ structures are displayed in **(b)** for E6 and in **(c)** for A3.

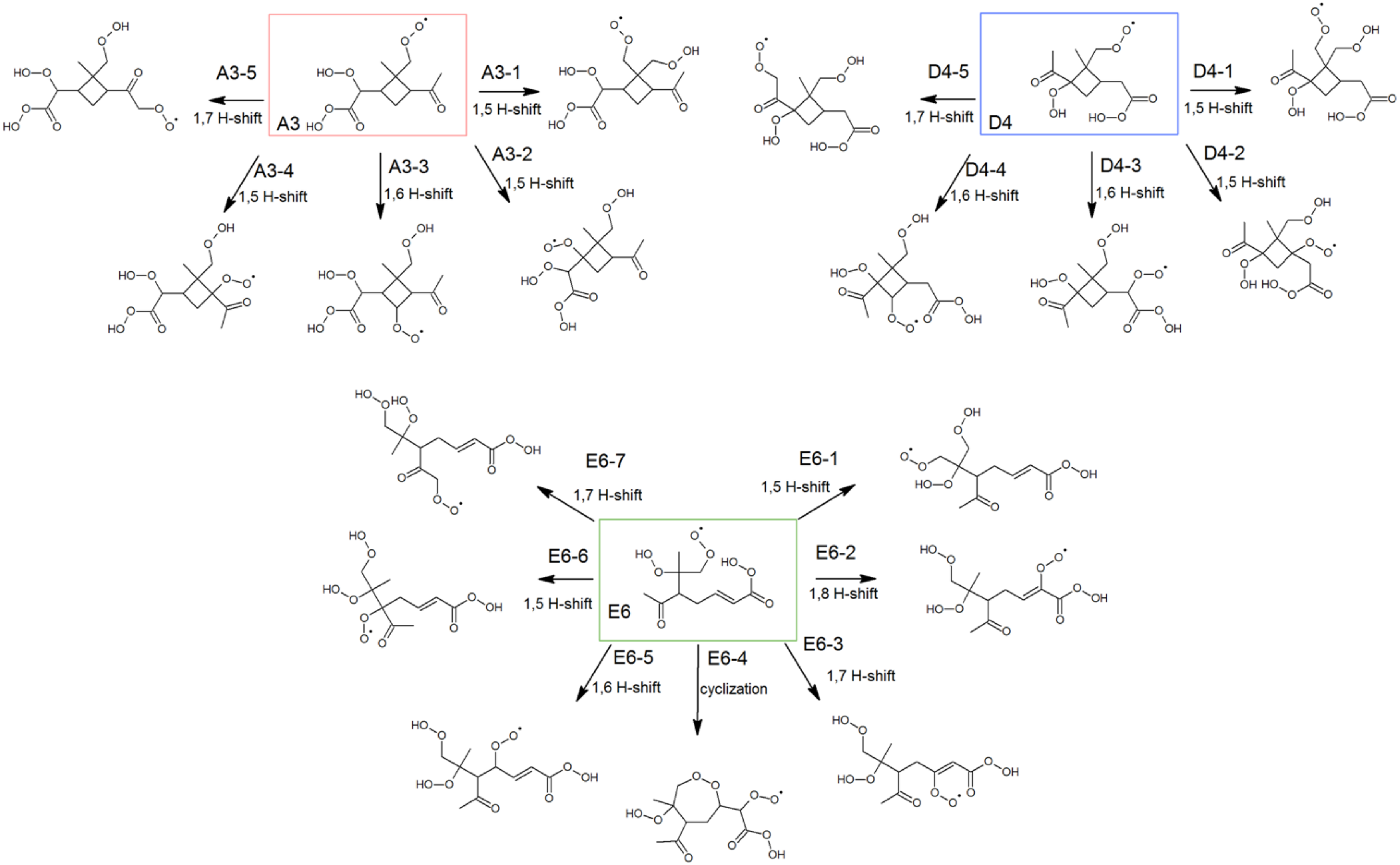

Supplementary Figure 11. Potential structures for the O_{10} peroxy radicals arising from the ozonolysis of α-pinene.

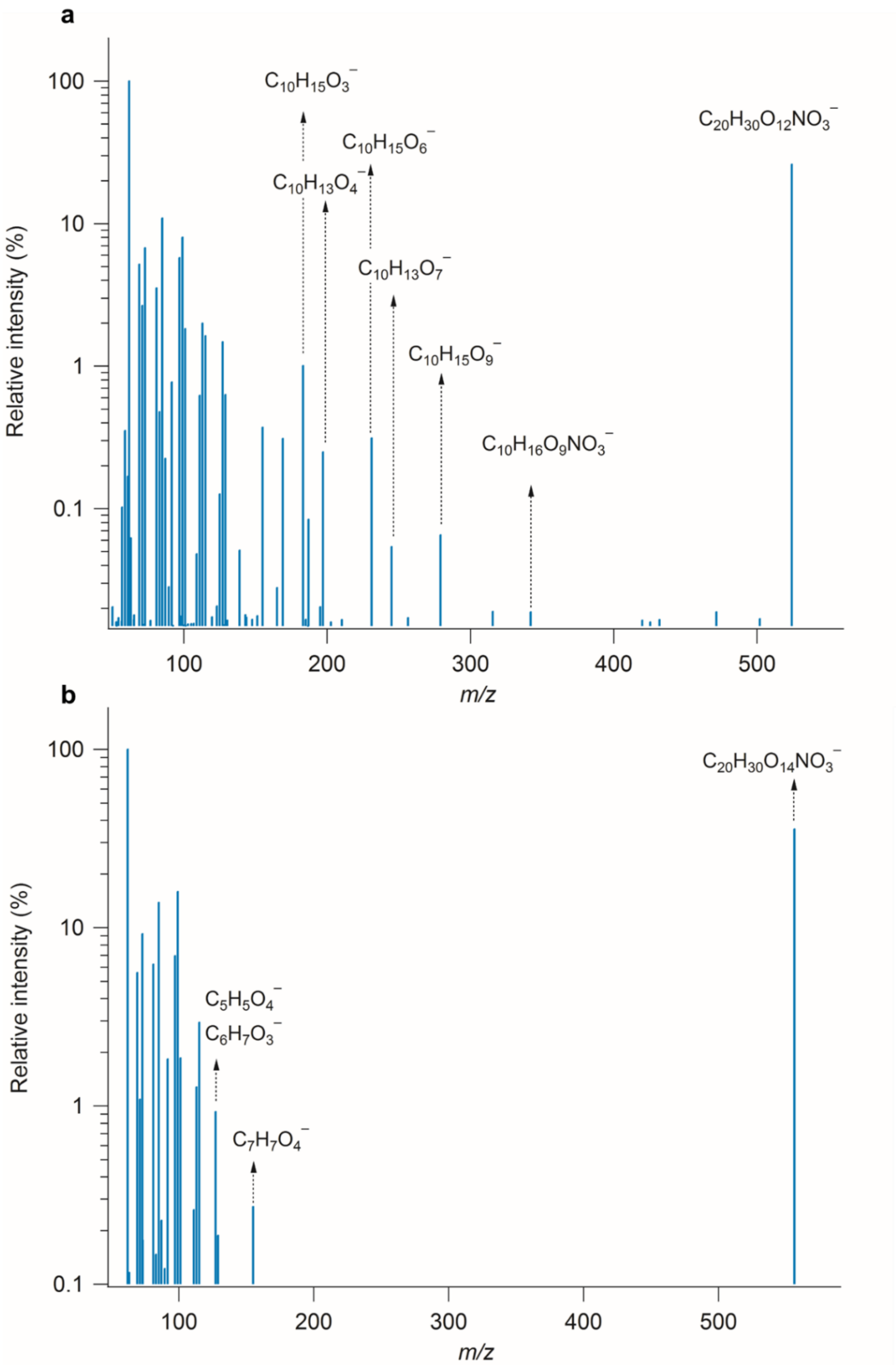

Supplementary Figure 12. MS/MS spectra of **a**, $C_{20}H_{30}O_{12}NO_3^-$ and **b**, $C_{20}H_{30}O_{14}NO_3^-$ dimers at NCE=5 from α-pinene ozonolysis.

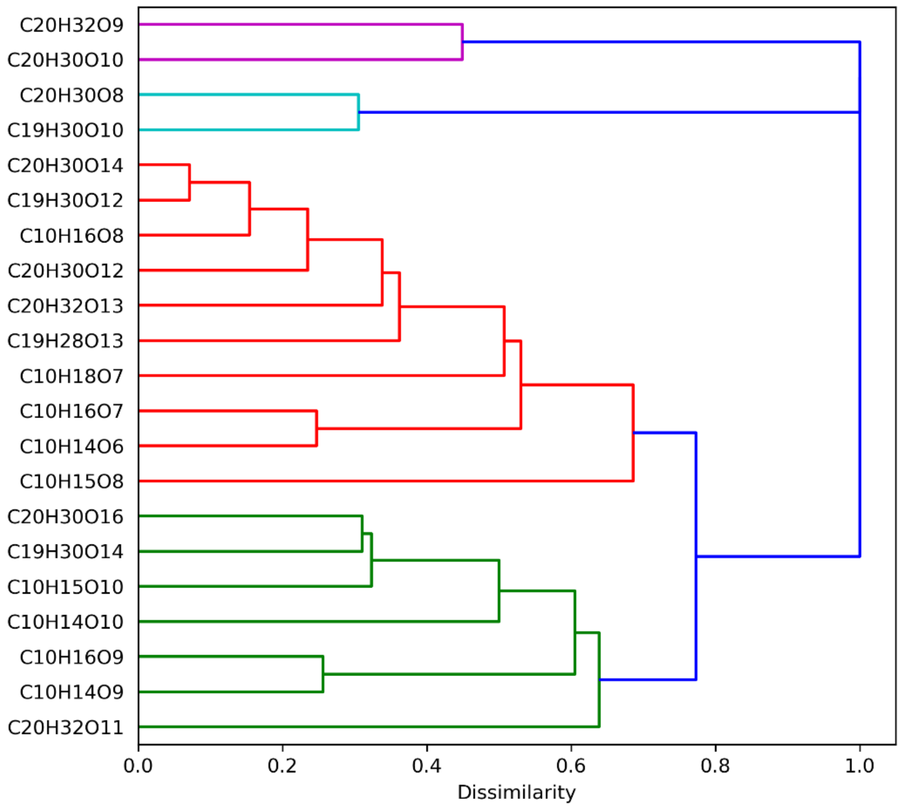

Supplementary Figure 13. Agglomerative hierarchical clustering of MS/MS product ions produced in limonene ozonolysis at NCE 5, excluding NO_3^- ions. Molecular formulae are shown on the y-axis. A similarity threshold of 0.7 is used to allocate clusters, represented by different colors. MS/MS spectra of $C_{10}H_{15}O_6$, $C_{18}O_{30}O_6$, $C_{18}O_{30}O_{10}$, $C_{19}H_{30}O_8$, $C_{19}H_{32}O_9$, and $C_{19}H_{28}O_9$ were distinct (i.e. dissimilarity > 0.7) from all other spectra and are therefore not shown.

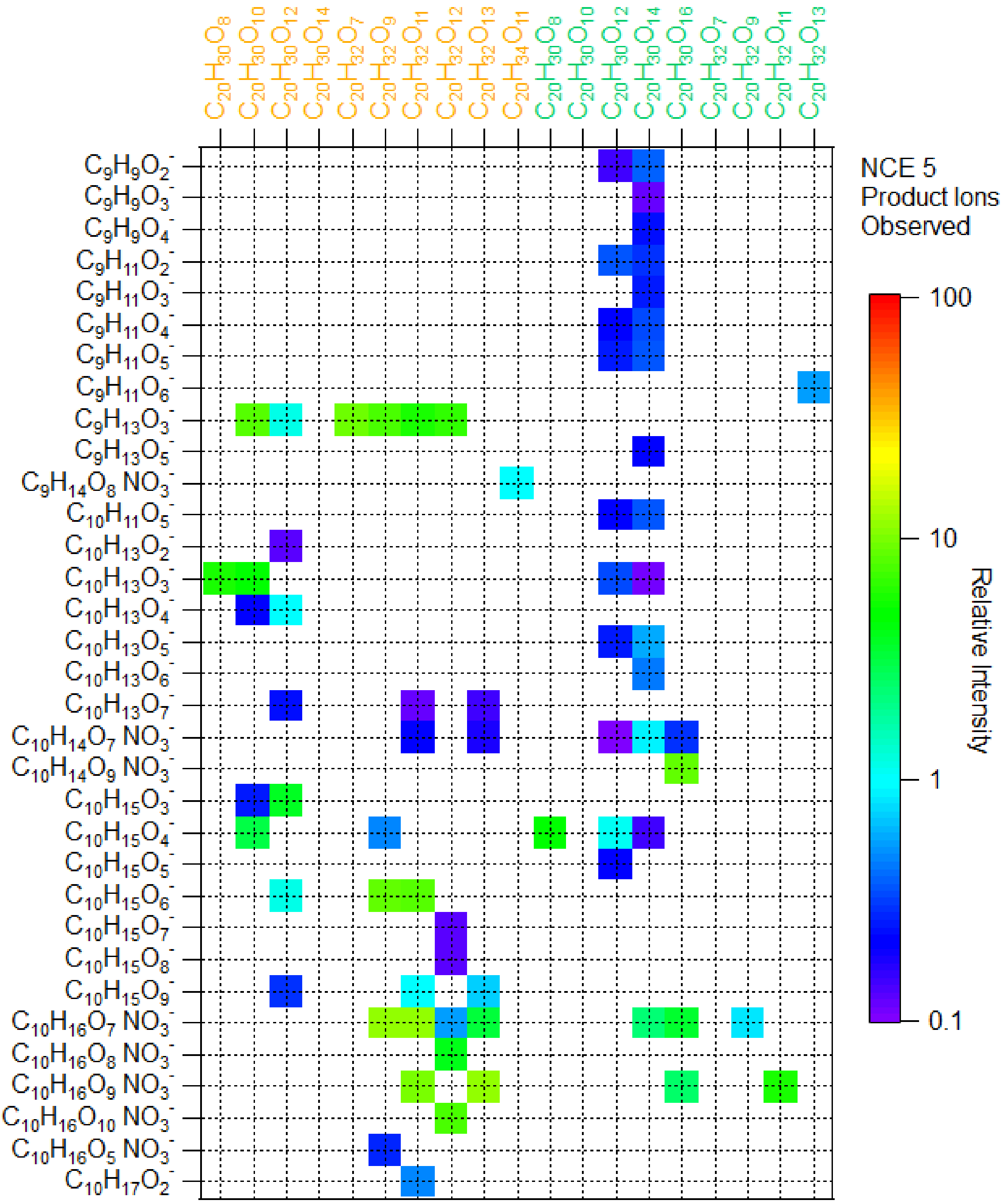

Supplementary Figure 14. Overview of the C_9 and C_{10} product ions observed in MS/MS spectra obtained at NCE 5 for NO_3^- adducts of limonene (green) and α-pinene (orange) ozonolysis dimers. Only ions with relative abundance above 0.1 are shown. Prevalence of $C_{10}H_{16}O_7$ NO_3^- among MS/MS of α-pinene $C_{20}H_{32}O_{9,11,12,13}$ point to the $C_{10}H_{15}O_8$ radical as a key participant in gas-phase reactions. $C_{10}H_{17}O_x$ produced from the oxidation of monoterpene by OH radicals. OH and HO_2 radicals are products of monoterpene ozonolysis.

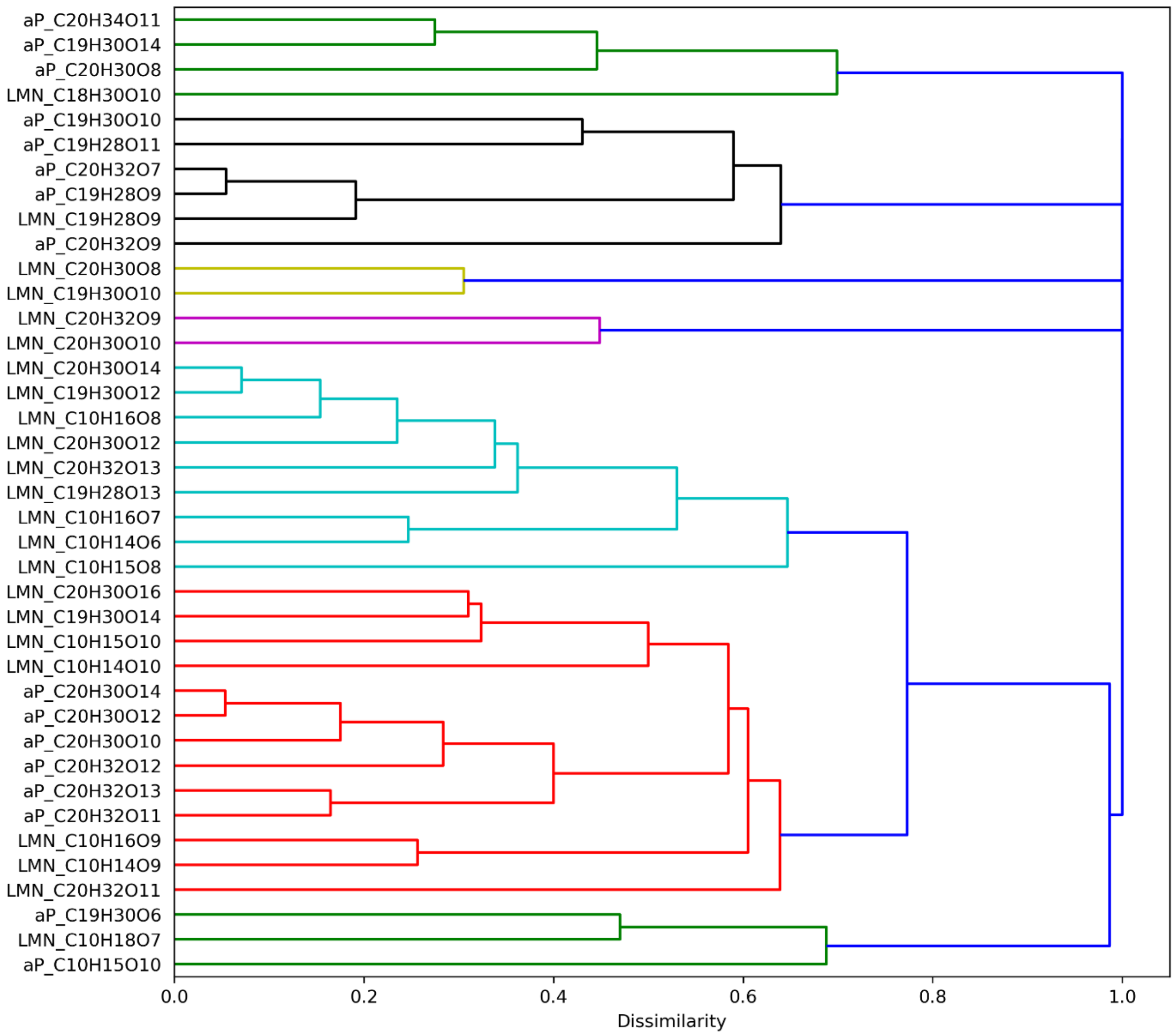

Supplementary Figure 15. Agglomerative clustering of MS/MS product ions of both limonene and α-pinene ozonolysis products at NCE 5, excluding NO_3^- ions. Molecular formulae are shown on the y-axis, where the prefixes indicate the VOC precursor: "LMN_" for limonene and "aP_" for α-pinene. A similarity threshold of 0.7 is used to allocate clusters, represented by different colors. Species that are distinct (i.e. dissimilarity > 0.7) from all other spectra are not shown, which includes aP_$C_{10}H_{15}O_8$ and aP_$C_{19}H_{30}O_8$, LMN_$C_{10}H_{15}O_6$, LMN_$C_{18}H_{30}O_6$, LMN_$C_{19}H_{30}O_8$, and LMN_$C_{19}H_{32}O_9$. Note that LMN_$C_{18}H_{30}O_{10}$ and LMN_$C_{19}H_{28}O_9$ were found to be distinct when only limonene ozonolysis product MS/MS spectra were analyzed.

Dimer Kinetics

The reaction rate coefficient RO_2 + $R'O_2$ → ROOR' + O_2 can be estimated using the method by Berndt et al. (2018)[4] if condensational loss and vapor wall loss are considered negligible,

$$k_{\mathrm{RO_2+R'O_2}} = \frac{3[\mathrm{ROOR'}]}{t[\mathrm{RO_2}][\mathrm{R'O_2}]} \qquad \text{Supplementary Equation 1}$$

where [ROOR'] is the concentration of the dimer, [RO_2] and [$R'O_2$] are the concentrations of the RO_2 radicals involved, and *t* is the reaction time, i.e. the flow tube residence time, roughly 60 s (the residence time was 51 second for most MS/MS runs). The condensational loss was negligible under all experimental conditions, where the total particle count (those ≥ 10_{nm} in diameter) remained below 100. The vapor wall loss lifetime of the flow tube, τ_{wall} is approximately 260 seconds, calculated using methods described by Palm et al., (2016),[5] assuming a mean gas diffusivity of 7 x 10^{-6} m^2 s^{-1}. Given a flow tube residence time, τ_{res} of ~60 seconds, approximately 18%, i.e. τ_{wall}^{-1} / (τ_{wall}^{-1} +τ_{res}^{-1}), of dimers formed are expected to be lost to the wall. Conversion of ion abundance from counts per second (cps) to molecule cm^{-3} was performed by applying a calibration factor of 2 x 10^9 molecule cm^{-3} cps^{-1} to the analyte ion signals normalized by the reagent ion signal (i.e. NO_3^- + $HNO_3NO_3^-$)). Intercomparison of mass spectrometer (TOF-MS) suggest that the nitrate ion-based chemistry sensitivity towards $C_{10}H_xO_6$ is roughly 2 orders of magnitude lower than other CI mass spectrometers (Riva et al., 2019). As a result, a correction factor of 100 is applied to the $C_{10}H_{15}O_6$ radical.

Supplementary Figures 16a and 16b illustrate the RO_2 radical and ROOR dimer dynamics during limonene and α-pinene ozonolysis. The RO_2 and dimer concentrations all increase with limonene concentration, as shown in Supplementary Figures 16a and 17c, as expected from increased production rate from limonene ozonolysis. In contrast, the somewhat counterintuitive decrease in O_{6-10} RO_2 radical concentrations with increasing α-pinene concentration shown in Supplementary Figures 16c and 17d, accompanied by increasing dimer concentrations, can be explained if the $C_{10}H_{15}O_4$ radical is a key participant in dimer formation, i.e., increased $C_{10}H_{15}O_4$ scavenges other RO_2 radicals to enhance dimer formation.

As discussed in the main text for limonene ozonolysis, the reaction of $C_{10}H_{15}O_8$ + $C_{10}H_{15}O_6$ contributes to the formation of $C_{20}H_{30}O_{12}$ and the reaction of $C_{10}H_{15}O_8$ + $C_{10}H_{15}O_8$ contributes to the formation of $C_{20}H_{30}O_{14}$. In addition, the observation of $C_{10}H_{14}O_9$ NO_3^- and $C_{10}H_{14}O_7NO_3^-$ as product ions of $C_{20}H_{30}O_{16}$ shown in Supplementary Figure 14 indicates that $C_{10}H_{15}O_8$ + $C_{10}H_{15}O_{10}$ contributes to $C_{20}H_{30}O_{16}$ formation. Using Supplementary Equation 1 and the correlation shown in Supplementary Figure 16e, we can estimate the RO_2 + $R'O_2$ → ROOR reaction rate coefficient, k_{dimer} for $C_{10}H_{15}O_8$ + $C_{10}H_{15}O_6$ → $C_{20}H_{30}O_{12}$ (6.07 x 10^{-11} $molecule^{-1}$ cm^3 s^{-1}), $C_{10}H_{15}O_8$ + $C_{10}H_{15}O_8$ → $C_{20}H_{30}O_{14}$ (1.08 x 10^{-9} $molecule^{-1}$ cm^3 s^{-1}), and $C_{10}H_{15}O_8$ + $C_{10}H_{15}O_{10}$ → $C_{20}H_{30}O_{12}$ (1.99 x 10^{-9} $molecule^{-1}$ cm^3 s^{-1}). The estimated k_{dimer} rates are fast, with $k_{C10H15O8+C10H15O8}$ and $k_{C10H15O8+C10H15O10}$ exceeding the kinetic limit, which may be due to processes such as CI inlet loss or an indication that other RO_2 + RO_2 reactions also contribute to the formation of $C_{20}H_{30}O_{12}$ and $C_{20}H_{30}O_{14}$. Nonetheless, our results suggests that dimer formation in the limonene system is rapid, with the reaction rate increasing with the oxygen content of the RO_2 radical as reported in an earlier study.

As shown in Supplementary Figure 12a for α-pinene ozonolysis, the formation of $C_{20}H_{30}O_{12}$ dimer is likely dominated by the reaction between $C_{10}H_{15}O_4$ and $C_{10}H_{15}O_{10}$ radicals. Similarly, the formation of $C_{20}H_{30}O_8$ and $C_{20}H_{30}O_{14}$ are likely driven by reactions between $C_{10}H_{15}O_4$ + $C_{10}H_{15}O_6$ and $C_{10}H_{15}O_4$ + $C_{10}H_{15}O_{12}$, respectively, under our experimental conditions.

Although $C_{10}H_{15}O_4$ is not observed, we can still calculate the k_{dimer} of $C_{10}H_{15}O_4 + C_{10}H_{15}O_6 \rightarrow C_{20}H_{30}O_8$, $C_{10}H_{15}O_4 + C_{10}H_{15}O_8 \rightarrow C_{20}H_{30}O_{10}$, and $C_{10}H_{15}O_4 + C_{10}H_{15}O_{10} \rightarrow C_{20}H_{30}O_{12}$ in relative terms using Supplementary Equation 2, derived from Supplementary Equation 1,

$$\frac{k_{C_{10}H_{15}O_4+C_{10}H_{15}O_x}}{k_{C_{10}H_{15}O_4+C_{10}H_{15}O_y}} = \frac{[C_{20}H_{30}O_{x+2}]}{[C_{10}H_{15}O_x]}\frac{[C_{10}H_{15}O_y]}{[C_{20}H_{30}O_{y+2}]} \quad \text{Supplementary Equation 2}$$

Relative to $k_{C10H15O4+C10H15O10}$ (set at 1), $k_{C10H15O4+C10H15O6}$ and $k_{C10H15O4+C10H15O8}$ (i.e. slopes in Supplementary Figure 16d) are 0.01 and 0.34 respectively. For the α-pinene ozonolysis system, the $RO_2 + RO_2$ reaction rate coefficient appears to increase with degree of oxidation of the RO_2 radical, similar to the limonene system and consistent with recent studies and parameterizations.[6,7]

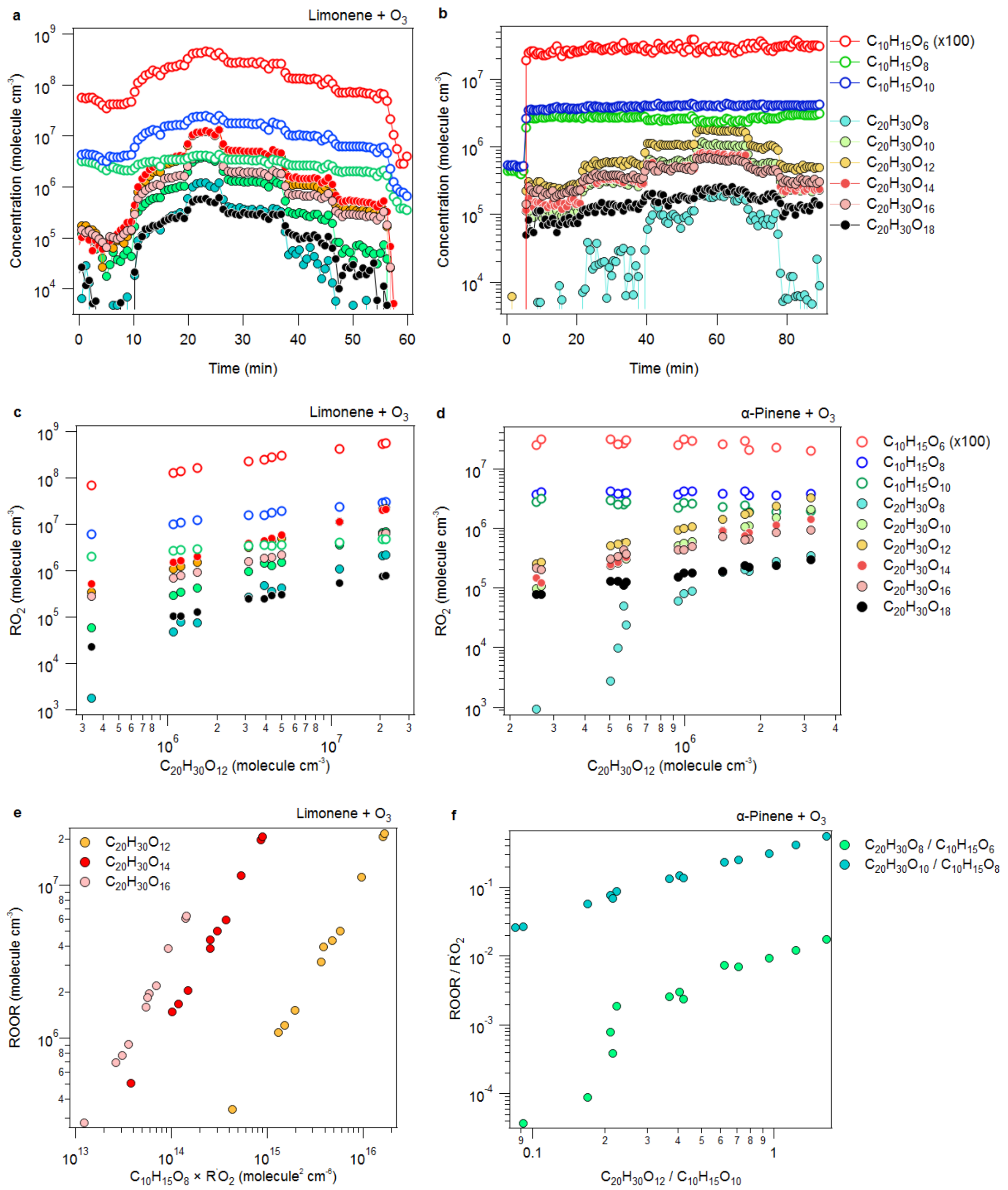

Supplementary Figure 16. (**a**) Time-series of limonene ozonolysis RO_2 radicals and dimer products as limonene concentration is varied between 25 and 253 ppbv in the presence of 20 ppbv O_3. (**b**) Time-series from α-pinene ozonolysis as α-pinene concentration is varied between 84 and 835 ppb in the presence of 40 ppbv O_3. Data shown are post-averaged over a 50-second window for (**a**) and (**b**). (**c**) Change in limonene ozonolysis RO_2 radicals and dimer product concentrations in relation to $C_{20}H_{30}O_{12}$ dimer. Data shown are post-averaged over a 4-minute window at each steady state. (**d**) Change in α-pinene ozonolysis RO_2 radicals and dimer product concentrations in relation to the $C_{20}H_{30}O_{12}$ dimer. For $C_{20}H_{30}O_8$ dimer, data below 10^5 molecule cm^{-3} are excluded from kinetic rate estimation due to concerns with nonlinearity at very low

concentrations.[8] (**e**) Correlation of [ROOR] with [RO_2] x [$R'O_2$], assuming $C_{10}H_{15}O_8$ as the precursor based on MS/MS results. The slope can be used to estimate the $RO_2 + R'O_2 \rightarrow ROOR + O_2$ reaction rate coefficient using Supplementary Equation 1. (f) Correlation of the ratio of $C_{20}H_{30}O_8$ / $C_{10}H_{15}O_6$ and $C_{20}H_{30}O_{10}$ / $C_{10}H_{15}O_8$ with that of $C_{20}H_{30}O_{12}$ / $C_{10}H_{15}O_{10}$. The slopes are used to calculate the relative k_{dimer} using Supplementary Equation 2.

Supplementary Methods
Online measurements
A Q Exactive Orbitrap mass spectrometer (Thermo Scientific, US) was used for online analysis of ozonolysis products. The instrument was operated in negative ion mode and externally calibrated using an aqueous solution of sodium acetate solution (2mM, Aldrich, > 99%). The source inlet capillary temperature was set at 35ºC. The MS/MS acquisition was done following two different settings described in Supplementary Table 1. For each precursor ion, the acquisition lasted at least 5 minutes. For low-signal intensity precursor ions, an acquisition time was extended to 10 minutes to improve the signal-to-noise ratio. The data were pre-averaged and analyzed using XCalibur 4.2 (Thermo Scientific) software.

Supplementary Table 1. Mass spectrometer parameters

Acquisition mode	Setting 1	Setting 2
RF level	60	100
Microscans	10	1
Automatic gain control (AGC)	$1x10^5$	$1x10^5$
Maximum injection time (ms)	3000	3000
Multi RF ratio	1.2	1.2
Acquisition time (min)	5 to 10	30
Masse resolution (at *m/z* 200)	140 000	70 000
NCE	2,5,7, and 10	2 and 5
CID	none	none
Isolation window (Da)	0.4	0.4

All flow tube experiments were operated at room temperature (24 ± 1 ^oC). Most experiments were run under dry conditions with a total flow rate of 21 liters per minute (LPM), which results in a residence time of approximately 51 second assuming plug flow inside the 18L flow tube. Specific experiments were run with lower total flow rate (15 LPM) and therefore longer residence time (72 seconds), or with moderate humidity (10-40%). Experimental conditions under which the MS/MS precursor ions were analyzed are detailed in Supplementary Tables 2-3.

Supplementary Table 2. Summary of MS/MS spectra investigated at NCE 2.

VOC	Precursor Ion	[VOC]	$[O_3]$	τ_{res}	RH	Settings
α-Pinene	$C_{10}H_{16}O_6\ NO_3^-$	214	30	51	0	2
α-Pinene	$C_{10}H_{16}O_7\ NO_3^-$	214	30	51	0	2
α-Pinene	$C_{10}H_{16}O_8\ NO_3^-$	214	30	51	0	2
α-Pinene	$C_{10}H_{16}O_9\ NO_3^-$	214	30	51	0	2
α-Pinene	$C_{10}H_{16}O_{10}\ NO_3^-$	214	30	51	0	2
α-Pinene	$C_{10}H_{15}O_8\ NO_3^{\bullet-}$	214	30	51	0	2
α-Pinene	$C_{10}H_{15}O_{10}\ NO_3^{\bullet-}$	214	30	51	0	2
Limonene	$C_{10}H_{14}O_7\ NO_3^-$	65	40	72	0	1
Limonene	$C_{10}H_{14}O_8\ NO_3^-$	217	28	51	0	2
Limonene	$C_{10}H_{14}O_9\ NO_3^-$	217	28	51	0	2
Limonene	$C_{10}H_{16}O_6\ NO_3^-$	227	30	51	0	2
Limonene	$C_{10}H_{16}O_7\ NO_3^-$	65	40	72	0	1
Limonene	$C_{10}H_{16}O_8\ NO_3^-$	227	30	51	0	2
Limonene	$C_{10}H_{16}O_9\ NO_3^-$	227	30	51	0	2
Limonene	$C_{10}H_{16}O_{10}\ NO_3^-$	227	30	51	0	2
Limonene	$C_{10}H_{15}O_8\ NO_3^{\bullet-}$	65	40	72	0	1
Limonene	$C_{10}H_{15}O_{10}\ NO_3^{\bullet-}$	65	40	72	0	1

"[VOC], ppbv": Input concentration of VOC in parts-per-billion by volume. "$[O_3]$, ppbv": Input concentration of O_3 in parts-per-billion by volume. "τ_{res}": Air flow residence time inside the flow tube assuming plug flow in seconds. "RH": Relative humidity in %

Supplementary Table 3. Summary of MS/MS spectra of α-pinene ozonolysis products investigated at NCE 5.

Precursor Ion	[VOC]	$[O_3]$	τ_{res}	RH	Setting
$C_{10}H_{15}O_8\ NO_3^{\bullet-}$	716	20	51	10	1
$C_{10}H_{15}O_{10}\ NO_3^{\bullet-}$	716	20	51	10	1
$C_{19}H_{28}O_9\ NO_3^-$	749	30	51	0	2
$C_{19}H_{28}O_{11}\ NO_3^-$	749	30	51	0	2
$C_{19}H_{30}O_6\ NO_3^-$	749	30	51	0	2
$C_{19}H_{30}O_8\ NO_3^-$	749	30	51	0	2
$C_{19}H_{30}O_{10}\ NO_3^-$	749	30	51	0	2
$C_{19}H_{30}O_{14}\ NO_3^-$	749	30	51	0	2
$C_{20}H_{30}O_8\ NO_3^-$	749	30	51	0	2
$C_{20}H_{30}O_{10}\ NO_3^-$	749	30	51	0	2
$C_{20}H_{30}O_{12}\ NO_3^-$	749	30	51	0	2
$C_{20}H_{30}O_{14}\ NO_3^-$	749	30	51	0	2
$C_{20}H_{32}O_7\ NO_3^-$	749	30	51	0	2
$C_{20}H_{32}O_9\ NO_3^-$	749	30	51	0	2
$C_{20}H_{32}O_{11}\ NO_3^-$	749	30	51	0	2
$C_{20}H_{32}O_{12}\ NO_3^-$	749	30	51	0	2
$C_{20}H_{32}O_{13}\ NO_3^-$	749	30	51	0	2
$C_{20}H_{34}O_{11}\ NO_3^-$	749	30	51	0	2

“[VOC], ppbv”: Input concentration of VOC in parts-per-billion by volume. “[O_3], ppbv”: Input concentration of O_3 in parts-per-billion by volume. “τ_{res}”: Air flow residence time inside the flow tube assuming plug flow in seconds. “RH”: Relative humidity in %

Supplementary Table 4. Summary of MS/MS spectra of limonene ozonolysis products investigated at NCE 5.

Precursor Ion	[VOC]	[O_3]	τ_{res}	RH	Setting
$C_{10}H_{14}O_7\ NO_3^-$	65	40	72	0	1
$C_{10}H_{14}O_7\ NO_3^-$	65	40	72	0	1
$C_{10}H_{14}O_9\ NO_3^-$	217	25	51	0	1
$C_{10}H_{14}O_{10}\ NO_3^-$	43	22	51	40	1
$C_{10}H_{15}O_6\ NO_3^{\bullet-}$	43	22	51	40	1
$C_{10}H_{15}O_8\ NO_3^{\bullet-}$	65	40	72	0	1
$C_{10}H_{15}O_{10}\ NO_3^{\bullet-}$	65	40	72	0	1
$C_{10}H_{16}O_8\ NO_3^-$	65	20	51	0	1
$C_{10}H_{16}O_9\ NO_3^-$	217	25	51	0	1
$C_{10}H_{18}O_7\ NO_3^-$	65	20	51	0	1
$C_{18}H_{30}O_6\ NO_3^-$	43	22	51	40	1
$C_{18}H_{30}O_{10}\ NO_3^-$	65	20	51	0	1
$C_{19}H_{28}O_9\ NO_3^-$	227	30	51	0	2
$C_{19}H_{28}O_{11}\ NO_3^-$	227	30	51	0	2
$C_{19}H_{28}O_{13}\ NO_3^-$	227	30	51	0	2
$C_{19}H_{30}O_8\ NO_3^-$	227	30	51	0	2
$C_{19}H_{30}O_{10}\ NO_3^-$	227	30	51	0	2
$C_{19}H_{30}O_{12}\ NO_3^-$	227	30	51	0	2
$C_{19}H_{30}O_{14}\ NO_3^-$	227	30	51	0	2
$C_{19}H_{32}O_9\ NO_3^-$	43	22	51	40	1
$C_{19}H_{32}O_9\ NO_3^-$	43	22	51	40	1
$C_{20}H_{30}O_8\ NO_3^-$	227	30	51	0	2
$C_{20}H_{30}O_{10}\ NO_3^-$	227	30	51	0	2
$C_{20}H_{30}O_{12}\ NO_3^-$	65	40	72	0	1
$C_{20}H_{30}O_{14}\ NO_3^-$	65	40	72	0	1
$C_{20}H_{30}O_{16}\ NO_3^-$	43	22	51	40	1
$C_{20}H_{32}O_7\ NO_3^-$	227	30	51	0	2
$C_{20}H_{32}O_9\ NO_3^-$	227	30	51	0	2
$C_{20}H_{32}O_{11}\ NO_3^-$	227	30	51	0	2
$C_{20}H_{32}O_{13}\ NO_3^-$	227	30	51	0	2

“[VOC], ppbv”: Input concentration of VOC in parts-per-billion by volume. “[O_3], ppbv”: Input concentration of O_3 in parts-per-billion by volume. “τ_{res}”: Air flow residence time inside the flow tube assuming plug flow in seconds. “RH”: Relative humidity in %

Offline measurements

Organic aerosol produced from α-pinene ozonolysis in a separate environmental chamber experiment were collected on particle filter. A 11 mm diameter circular punch of the filter was cut into small pieces and extracted with 500 µl of a 95:5 (by volume) water:methanol solution for 20 minutes. At the end of the extraction period, the solution was filtered through a 0.2 µm syringe filter to rid of solid debris. The extract was separated by high-performance liquid

chromatography on a reversed-phase column (Thermo Scientific Accucore C_{18}, 2.6 µm, 150 x 2.1 mm), ionized in the negative and positive modes using an electrospray ionization source (HESI-II Probe, Thermo Fisher Scientific), and measured with a high-resolution hybrid quadrupole-Orbitrap mass spectrometer (Q Exactive Focus, Thermo Fisher Scientific). An injection volume of 2 µl, a flow rate of 400 µl/min, and a column oven temperature of 50 °C were applied. A gradient of ultrapure water (Optima LC-MS, Fisher Chemical) with 0.1 % formic acid (>99.0%., Fisher Chemicals) (eluent A) and methanol (Optima LC-MS, Fisher Chemicals) (eluent B) applied for separation. The gradient started with 1 % eluent B (0-2 min), linearly increasing to 99 % B (2-12 min), staying at 99 % B (12-16 min), before re-equilibration (total gradient length of 20 min). The ion source settings were as follows in both polarities: 3.5 kV spray, 50 psi sheath gas, 13 psi auxiliary gas, 400 °C gas temperature, and 250 °C capillary temperature. The spectra were recorded in full scan MS with data-dependent tandem mass spectrometry (ddMS/MS). The scan range in full MS was *m/z* 50-750 with a resolution of 70 000 at *m/z* 200. The MS-data were recorded in centroid mode. The sodium-dimer of formic acid $(CHO_2)_2Na^-$ at *m/z* 112.9856 was used as the internal standard for lock-mass calibration in the negative mode. Protonated dimethylformamid (or the methanol/acetonitrile cluster) $C_3H_7NOH^+$ at *m/z* 74.0600 and diisooctlyphthalate $C_{24}H_{38}O_4H^+$ at *m/z* 391.2843 were used as the internal standards for lock-mass calibration in the positive mode.

Supplementary References

1. Kurtén, T. *et al.* Computational study of hydrogen shifts and ring-opening mechanisms in α-pinene ozonolysis products. *J. Phys. Chem. A* **119**, 11366–11375 (2015).
2. Rissanen, M. P. *et al.* Effects of chemical complexity on the autoxidation mechanisms of endocyclic alkene ozonolysis products: from methylcyclohexenes toward understanding α-pinene. *J. Phys. Chem. A* **119**, 4633–4650 (2015).
3. P. Demarque, D., M. Crotti, A. E., Vessecchi, R., C. Lopes, J. L. & P. Lopes, N. Fragmentation reactions using electrospray ionization mass spectrometry: an important tool for the structural elucidation and characterization of synthetic and natural products. *Nat. Prod. Rep.* **33**, 432–455 (2016).
4. Berndt, T. *et al.* Accretion product formation from self- and cross-reactions of RO_2 radicals in the atmosphere. *Angew. Chem. Int. Ed.* **57**, 3820–3824 (2018).
5. Palm, B. B. *et al.* In situ secondary organic aerosol formation from ambient pine forest air using an oxidation flow reactor. *Atmos. Chem. Phys.* **16**, 2943–2970 (2016).
6. Molteni, U. *et al.* Formation of highly oxygenated organic molecules from α-pinene ozonolysis: chemical characteristics, mechanism, and kinetic model development. *ACS Earth Space Chem.* **3**, 873–883 (2019).
7. Schervish, M. & Donahue, N. M. Peroxy radical chemistry and the volatility basis set. *Atmos. Chem. Phys.* **20**, 1183–1199 (2020).
8. Riva, M. *et al.* Capability of CI-orbitrap for gas-phase analysis in atmospheric chemistry: a comparison with the CI-APi-TOF technique. *Anal. Chem.* **92**, 8142–8150 (2020).
